# Rising Risk of Subsequent Primary Cancers Among US Cancer Survivors, 2000–2021

**DOI:** 10.1002/cam4.71778

**Published:** 2026-04-06

**Authors:** Hui G. Cheng, Livingstone Aduse‐Poku, Oxana Palesh, Susan Hong

**Affiliations:** ^1^ Massey Comprehensive Cancer Center Virginia Commonwealth University Richmond Virginia USA

**Keywords:** cancer survivors, epidemiology, multiple primary cancers, SEER, standardized incidence ratio, survivorship, time trends

## Abstract

**Background:**

As cancer survival improves, the population at risk for subsequent primary cancers (SPCs) is growing rapidly. Yet, contemporary trends in SPC incidence remain undercharacterized, limiting the development of targeted surveillance and survivorship strategies.

**Objective:**

To evaluate long‐term trends in SPC risk among US cancer survivors and identify high‐risk subgroups by cancer type and demographic characteristics.

**Design, Setting, and Participants:**

This retrospective cohort study used data from 17 SEER registries and included over 6 million individuals diagnosed with an index primary cancer between 2000 and 2021.

**Main Outcomes and Measures:**

Standardized incidence ratios (observed‐to‐expected ratios, OERs) and Cox proportional hazards models were used to assess SPC trends among male and female cancer survivors. Analyses were stratified by cancer site, latency between index and SPC, stage, age, and race/ethnicity.

**Results:**

SPC risk increased substantially from 2000 to 2020. Among men, OERs rose from 0.95 (95% CI = 0.94, 0.97) to 1.75 (95% CI = 1.70, 1.80); among women, from 1.23 (95% CI = 1.21, 1.24) to 1.76 (95% CI = 1.70, 1.83). The highest SPC incidence occurred within 6 months of the index diagnosis. Variations were observed across demographic and cancer‐related characteristics.

**Conclusions and Relevance:**

The rising burden of SPCs highlights a critical need in survivorship care. These findings support the need for updated, risk‐stratified surveillance protocols and inform national cancer control strategies aimed at reducing long‐term morbidity among survivors.

**Study Type:**

Cohort study.

## 1 Introduction

The global population of cancer survivors is rapidly expanding, driven by aging demographics and improved survival following advances in early detection and treatment [1]. Worldwide, cancer cases are projected to rise 77% between 2022 and 2050 [1]. In the United States (US), the survivor population is expected to grow by 22% over the next decade from 18 million in 2025 to more than 22 million by 2035 [2]. This growing cancer survivor population faces unique long‐term health challenges that extend beyond acute treatment outcomes. One such challenge is the development of subsequent primary cancers (SPC), distinct malignancies arising in different sites and/or of different histologic or morphologic features in the same individual [3]. Prior studies estimated that up to 18% of cancer survivors might develop SPCs within 20 years of the initial cancer diagnosis—a rate substantially higher than that in the general population [4, 5]. As survivor populations grow, understanding trends in SPCs is essential not only for public health and clinical planning but also for the development of evidence‐based surveillance protocols tailored to cancer survivors. Recent analyses have shown that SPCs account for an increasing portion of new cancer diagnoses: the proportion of SPCs more than doubled from 10% in 1975 to 21% in 2017, underscoring the significance of monitoring and addressing SPCs in survivorship care [6]. Identifying high‐risk subgroups based on characteristics of the index cancer and demographics (such as age and race/ethnicity) is critical to guide resource allocation, reduce disparities, and optimize survivorship care pathways.

Several factors contribute to the elevated SPC risk among survivors. Tumor‐related characteristics—such as the site, histology, and stage of the index cancer—shape both underlying biology and the likelihood of intensive diagnostic evaluation, which may influence SPC detection [5, 7]. Treatment modalities, including radiation, chemotherapy, and hormonal therapies, are well‐established contributors to therapy‐related malignancies and may confer long‐term risk that varies by treatment era and regimen [8, 9, 10, 11]. Intrapersonal factors such as age, comorbidities, smoking, obesity, and other lifestyle exposures also play a critical role, as many risk factors for primary cancers similarly predispose survivors to new malignancies [12, 13, 14, 15]. These diverse influences underscore that SPC risk is shaped by a complex interplay of clinical, demographic, and behavioral factors [16]. As more individuals survive cancer, these risks may evolve. A prior study documented a stable trend from 1973 to 1994 followed by a slight uptick in SPC risk during 1995–2000 [5], but more recent data are lacking.

In this study, we aim to provide a contemporary overview of SPC occurrence in the United States, offering foundational estimates to support strategic planning, clinical guideline updates, and resource allocation. Specifically, we estimate time trends in SPC risk among cancer survivors relative to the general population using a large cohort of individuals diagnosed between 2000 and 2021 in the United States. Given the complexity and heterogeneity of SPC risk, stratified analyses can help characterize subgroup‐specific risks and to inform tailored survivorship care. Differences by age, race/ethnicity, cancer site, stage, and latency since diagnosis can guide tailored surveillance or prevention. Despite the clinical importance of these patterns, contemporary population‐based estimates remain limited. Therefore, we conducted stratified analyses by cancer characteristics (e.g., latency, site, and stage of the index cancer) and demographic factors. Findings from this study will provide critical information for surveillance for new cancers, a significant evidence gap in cancer survivorship research [16].

## 2 Methods

### 2.1 Study Design and Data Source

In this retrospective cohort study, we used data from 17 Surveillance Epidemiology and End Results (SEER) registries (excluding Alaska) from 2000 to 2021, representing approximately 26.5% of the US population [17]. SEER captures all cancers diagnosed within the designated geographic area, making it a comprehensive, population‐based source of cancer data in the United States [18]. Trained registrars collect data from hospitals, outpatient clinics, radiology departments, doctors' offices, laboratories, surgical centers, or other clinical providers. Information on SPC was obtained by tracking individuals across multiple cancer diagnoses within a SEER registry.

Sociodemographic variables available in the public use version of SEER include age at diagnosis, sex, race, ethnicity (derived using the NAACCR Hispanic Identification Algorithm), and county‐level socioeconomic indicators (e.g., % living below the federal poverty level, % with high‐school education, and median family income). Clinical variables include cancer site and histology (ICD‐O‐3 topography and morphology codes), behavior code, year of diagnosis, tumor grade, and stage. Detailed definitions and coding specifications for SEER sociodemographic and clinical variables are available in the SEER*Stat documentation (https://seer.cancer.gov/data‐software/documentation/seerstat/nov2024/).

In this study, we included all incident malignant tumors recorded in SEER (ICD‐O‐3 behavior code = ‘malignant’) across all primary cancer sites, excluding cases identified solely through death certificate or autopsy reports [19]. Both solid tumors and hematologic malignancies were included. We restricted the cohort to first primary cancers diagnosed from 2000 onward because complete multiple primary coding before 2000 is not available for all registries included in the SEER‐17 database, which limits reliable identification of earlier diagnoses.

### 2.2 Definitions and Statistical Analysis

SPCs were defined using the SEER Multiple Primary and Histology Coding Rules [20, 21]. The first SEER‐recorded cancer was designated as the index cancer, and all subsequent primary cancers were classified as SPCs. Individuals remained in the cohort unless lost to follow‐up, deceased, or end of study, contributing person‐time throughout their follow‐up. To reduce potential misclassification, we excluded SPC diagnosed within 2 months after the index cancer following recommendations from SEER [22, 23]. To examine residual misclassification, we conducted a sensitivity analysis to exclude SPC diagnosed within 6 months after the index cancer.

To estimate the risk of developing SPC among cancer survivors relative to the general population, we focused on the observed‐to‐expected ratio (OER) [5, 24], given by the following:

$$\mathrm{OER}=\frac{\sum_{k=1}^{M}D_{k}}{\sum_{k=1}^{M}t_{k}\lambda_{k}^{*}}$$

where *M* is the number of strata defined by sex, age, race/ethnicity, and calendar year; $D_{k}$ is the number of observed SPC cases that the cohort contribute to the k^th^ stratum; $t_{k}$ represents the person‐time of that stratum (i.e., person‐years accrued from the time of the index cancer to incidence of SPC or censoring), and $\lambda_{k}^{*}$ represents the incidence in the general population for the *k*th stratum. SEER general‐population incidence was calculated as the number of incident first primary cancers occurring in the SEER registries divided by the corresponding population denominators of the area derived from US Census population estimates [25]. Thus, the OER for a given calendar year is a type of standardized incidence ratio comparing SPC incidence among survivors diagnosed in that year with the expected incidence in the general population. An OER > 1 indicates higher‐than‐expected incidence of SPC among survivors compared to the general population.

To assess temporal trends, we estimated OER for each calendar year of index cancer diagnosis and plotted OERs over time. We evaluated temporal patterns using Joinpoint regression, which identifies inflection points and estimates Annual Percent Change (APC) in OERs across segments [26, 27]. This approach allowed us to quantify how SPC risk changed over time and whether increases accelerated in recent years. An APC > 0 indicates an increasing trend and vice versa. To allow sufficient follow‐up time, index cancer diagnosed in 2021 was excluded.

We then further stratified by selected cancer characteristics (i.e., the latency of cancers, the site of the index cancer, and the stage of the index cancer) and demographic characteristics (age at diagnosis of the index cancer and race/ethnicity). Except for analyses stratified by index cancer site, all malignant sites were pooled in the analyses. In this study, we categorized age into 0–14 years, 15–39 years, 40–49 years, 50–64 years, 65–84 years, and ≥ 85 years to align with childhood, adolescence and young adulthood, and adulthood. We further divided adulthood into three period given the inverse U‐shaped curve of cancer incidence [3]. We created five mutually exclusive race/ethnicity categories: Hispanic (any race), non‐Hispanic (NH) White, NH Black, NH Asian/Pacific Islander, and NH American Indian/Alaska Native. The stage of cancer was assigned using the SEER summary stage; analyses stratified by stage were restricted to cases diagnosed in 2004 and later to ensure consistency.

To complement the OER analyses, we used Cox proportional hazards models to estimate the hazard of developing an SPC as a function of the year of diagnosis of the index cancer. Calendar year was modeled categorically, with 2000 as the reference, allowing direct comparison of SPC risk among survivors diagnosed in later years. A hazard ratio > 1 indicates higher SPC risk relative to survivors diagnosed in 2000. In essence, OERs quantify population‐relative SPC incidence, whereas Cox models estimate temporal differences in SPC risk within the survivor cohort. Together, they provide a comprehensive assessment of changing SPC risk over time.

All analyses were stratified by males and females. Because previous studies have raised concerns about the potential misclassification of recurrent breast cancer as SPC under SEER definitions, we conducted a sensitivity analysis that restricted the sample to tumors meeting the international rule for primary cancers [28, 29]. SEER*Stat does not directly implement the International Agency for Research on Cancer/International Association of Cancer Registries rules, but the “Primary by International Rules” variable provides an approximation of these criteria. Analyses were conducted from December 2024 to August 2025 using SEER*stat, Joinpoint (version 5.4), and Stata (MP 18.0) [30].

## 3 Results

### 3.1 Sample Description

The study cohort comprised 6,110,519 individuals diagnosed with an index cancer between 2000 and 2020. The sex distribution was balanced, with 49% females (*n* = 2,995,233) and 51% males (*n* = 3,115,286). Racial/ethnic composition was 11% Hispanics, 71% NH white, 10% NH Black, 7% NH Asian and NH Pacific Islander, and 0.5% NH American Indian/Alaskan Native. Forty‐four percent of index cancers were diagnosed between ages 65 and 84 years, 33% between 50 and 64, 9% between 40 and 49, 6% between 15 and 39, 6% at age 85 or older, and 1% before age 15.

During the follow‐up, 619,783 cancer survivors received 707,429 subsequent primary cancer diagnoses. Fifteen percent of SPCs occurred within the first year of index cancer diagnosis (8% within 6 months, 7% between 6–12 months); 38% occurred between 1 and < 5 years, 28% between 5 and < 10 years, and 20% at ≥ 10 years. The top five index cancer sites accounted for over 60% of cases in both sexes. For females, the top five sites were breast (32.0%), lung/bronchus (10.3%), colorectal (9.8%), uterine corpus (6.6%), and melanoma (4.1%). For males, the top five sites were prostate (29.8%), lung/bronchus (10.9%), colorectal (9.4%), urinary bladder (6.3%), and melanoma (5.2%). The most common subsequent primary cancers were breast (20.7%), lung/bronchus (18.6%), and colorectal (7.3%) in females; and lung/bronchus (17.3%), melanoma (14.2%), prostate (9.6%), and colorectal (6.6%) in males.

Table 1 presents the distribution of SPC sites by index cancer and by latency intervals. Among women, same‐site clustering was prominent among survivors of breast cancer, lung and bronchus cancer, and skin melanoma. Overall breast, colon and rectum, and lung and bronchus were most common SPC sites. Among men, strong same‐site clustering was observed for lung cancer, urinary bladder cancer, and skin melanoma. Overall, most common SPC sites were prostate, colon and rectum, lung and bronchus, urinary bladder, and skin melanoma. Patterns also varied by latency. Same‐site or adjacent‐site SPCs tended to account for a larger share of SPCs within 6 months of the index cancer diagnosis for a few sites (e.g., same‐site skin melanoma and prostate cancer after urinary bladder cancer), whereas a bimodal pattern with peaks at early and late SPCs was observed for other sites such as same‐site SPC after breast and lung and bronchus cancer in women.

**TABLE 1:** Proportion (%) of subsequent primary cancers (SPC) by index primary cancer by latency intervals.

SPC site	Total	Latency				
		2–5 months	6–12 months	13–59 months	60–119 months	120+ months
Female						
*Index cancer site = breast*						
Breast	35%	43%	26%	27%	37%	41%
Lung and bronchus	13%	15%	11%	13%	13%	12%
Colon and rectum	8%	6%	10%	10%	7%	6%
Corpus uteri	6%	5%	6%	6%	6%	5%
Melanoma of the skin	4%	2%	4%	4%	3%	3%
*Index cancer site = colon and rectum cancer*						
Breast	21%	17%	21%	19%	23%	24%
Colon and rectum	19%	24%	20%	24%	15%	13%
Lung and bronchus	14%	15%	13%	14%	16%	14%
Corpus Uteri	6%	5%	6%	6%	7%	6%
Non‐hodgkin lymphoma	4%	4%	3%	3%	4%	4%
*Index cancer site = uterine*						
Breast	30%	20%	29%	30%	31%	30%
Colon and rectum	12%	8%	12%	12%	12%	13%
Lung and bronchus	11%	8%	11%	11%	11%	10%
Non‐hodgkin lymphoma	4%	4%	3%	4%	4%	5%
Melanoma of the skin	4%	1%	3%	3%	4%	4%
*Index cancer site = lung and bronchus*						
Lung and bronchus	49%	40%	31%	47%	57%	55%
Breast	13%	13%	14%	14%	12%	12%
Colon and rectum	6%	8%	9%	6%	4%	6%
Urinary bladder	3%	3%	5%	3%	3%	2%
Pancreas	3%	3%	5%	3%	2%	2%
*Index cancer site = skin melanoma*						
Melanoma of the skin	35%	46%	45%	37%	32%	30%
Breast	21%	16%	17%	20%	22%	23%
Lung and bronchus	8%	8%	7%	7%	8%	8%
Colon and rectum	5%	4%	4%	5%	5%	5%
Corpus uteri	4%	3%	3%	4%	5%	4%
Male						
*Index cancer site = prostate*						
Lung and bronchus	17%	15%	18%	18%	18%	16%
Urinary bladder	13%	15%	9%	12%	12%	14%
Colon and rectum	11%	11%	13%	13%	11%	10%
Melanoma of the skin	8%	5%	7%	8%	9%	9%
Kidney	6%	15%	9%	6%	6%	5%
Non‐hodgkin lymphoma	6%	8%	6%	6%	6%	6%
*Index cancer site = colon and rectum cancer*						
Prostate	22%	23%	23%	21%	22%	21%
Colon and rectum	18%	22%	19%	23%	14%	11%
Lung and bronchus	14%	13%	13%	13%	15%	14%
Urinary bladder	7%	5%	6%	7%	8%	9%
Melanoma of the skin	4%	2%	4%	4%	4%	5%
*Index cancer site = lung and bronchus*						
Lung and bronchus	39%	27%	24%	39%	48%	45%
Prostate	13%	12%	14%	14%	11%	10%
Urinary bladder	8%	7%	8%	8%	7%	8%
Colon and rectum	6%	8%	9%	6%	5%	5%
Kidney	3%	7%	6%	3%	2%	2%
*Index cancer site = urinary bladder*						
Prostate	29%	62%	43%	23%	18%	16%
Urinary bladder	17%	13%	23%	20%	15%	12%
Lung and bronchus	16%	7%	9%	17%	20%	22%
Colon and rectum	5%	2%	4%	6%	6%	6%
Melanoma of the skin	3%	1%	2%	3%	4%	5%
*Index cancer site = skin melanoma*						
Melanoma of the skin	38%	42%	44%	38%	35%	35%
Prostate	20%	15%	17%	20%	21%	20%
Lung and bronchus	7%	6%	5%	7%	7%	7%
Urinary bladder	5%	3%	5%	5%	5%	5%
Colon and rectum	4%	4%	4%	5%	5%	4%

*Note:* Data from the United States Surveillance, Epidemiology, and End Results Program 2000–2020. Percentages are within index cancer–specific SPCs (i.e., column %). The top 5 SPC sites are shown.

### 3.2 Overall Trends

**FIGURE 1:**
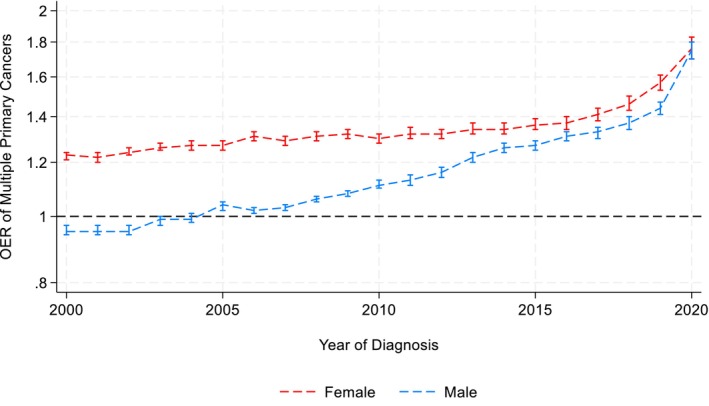
Observed/expected ratio (OER) of subsequent primary cancers among males and females over time. Data from the United States Surveillance, Epidemiology, and End Results Program 2000–2020. Brackets represent the 95% confidence interval of each estimate. For females, the Joinpoint regression identified one significant inflection points in 2017. From 2000 to 2017, OER increased with an APC of 0.7% (95% CI: [0.5%, 0.8%], *p* < 0.001). Between 2017 and 2020, OER rose with an APC of 7.5% (95% CI: [4.0%, 12.1%]). For males, the Joinpoint regression identified two significant inflection points in 2007 and 2018. From 2000 to 2007, OER increased with an Annual Percent Change (APC) of 1.3% (95% CI: [0.1%, 1.7%], *p* = 0.041). Between 2007 and 2018, APC was 2.6% (95% CI: [2.2%, 3.1%]). From 2018 to 2020, APC was 10.5% (95% CI: [4.6%, 13.2%]).

OERs for SPC increased steadily for both sexes (Figure 1; Table S1‐1). Female survivors consistently had higher SPC incidence than the general population, with OER rising from 1.23 (95% CI: 1.21, 1.24) in 2000 to 1.76 (95% CI: 1.70, 1.83) in 2020. Male survivors initially had lower SPC incidence than the general population (OER = 0.95; 95% CI: 0.94, 0.97 in 2000), but this rose above 1 in 2005 and reached 1.75 (95% CI: 1.70, 1.80) by 2020. For females, Joinpoint regression found a modest rise in OER from 2000 to 2017 with an annual increase of 0.7% (95% CI: 0.5%, 0.8%) and then accelerated between 2017 and 2020 with an annual increase of 7.5% (95% CI: 4.0%, 12.1%). This progressive increase is seen for males as well, annual change increased from 1.3% (95% CI: 0.1%, 1.7%) during 2000–2007 to 2.6% (95% CI: 2.2%, 3.1%) during 2007–2018 and 10.5% (95% CI: 4.3%, 13.2%) from 2018 to 2020. Sensitivity analysis excluding SPCs diagnosed within 6 months of the index cancer showed similar increasing trends (OER increased from 1.22 (95% CI = 1.20, 1.24) in 2000 to 1.47 (95% CI = 1.41, 1.53) in 2020 among women, and from 0.94 (95% CI = 0.92, 0.95) in 2000 to 1.48 (95% CI = 1.42, 1.53) in 2020 among men; Table S1‐2).

Cox regression results aligned with the OER trends, indicating an increasing hazard of developing SPCs over time (Table 2). Compared to female cancer survivors diagnosed in 2000, those diagnosed in later years had progressively higher risks of SPC. For instance, the hazard increased from 4% higher in 2002 (HR = 1.04; 95% CI: 1.01, 1.06) to 40% higher in 2020 (HR = 1.40; 95% CI: 1.35, 1.46). Similarly, the hazard of developing SPC rose from 5% higher among males diagnosed in 2003 (HR = 1.05; 95% CI: 1.03, 1.07) to 51% higher among those diagnosed in 2020 (HR = 1.51; 95% CI: 1.46, 1.56), relative to men diagnosed in 2000. Adjustment for age, race/ethnicity, cancer site, and stage of the index cancer had minimal impact on estimates for women, but strengthened the observed associations among men (primarily driven by the site of cancer).

**TABLE 2:** Estimated hazard ratio (95% CI) of subsequent primary cancers by year from cox regression (Reference year = 2000).

Year	Females		Males	
	Model 1	Model 2	Model 1	Model 2
2000	Reference			
2001	1.01 (0.98,1.03)	1.01 (0.98,1.03)	1.01 (0.99,1.03)	**1.02 (1.00,1.04)**
2002	**1.04 (1.01,1.06)**	**1.04 (1.02,1.06)**	1.01 (0.99,1.03)	**1.04 (1.02,1.06)**
2003	**1.05 (1.03,1.08)**	**1.05 (1.03,1.08)**	**1.05 (1.03,1.07)**	**1.07 (1.05,1.09)**
2004	**1.07 (1.05,1.09)**	**1.05 (1.03,1.07)**	**1.07 (1.05,1.09)**	**1.07 (1.05,1.09)**
2005	**1.08 (1.06,1.11)**	**1.06 (1.04,1.09)**	**1.11 (1.08,1.13)**	**1.11 (1.09,1.13)**
2006	**1.12 (1.10,1.15)**	**1.11 (1.08,1.13)**	**1.10 (1.08,1.12)**	**1.13 (1.11,1.16)**
2007	**1.11 (1.09,1.14)**	**1.10 (1.08,1.13)**	**1.11 (1.09,1.13)**	**1.16 (1.13,1.18)**
2008	**1.13 (1.10,1.15)**	**1.12 (1.09,1.14)**	**1.14 (1.11,1.16)**	**1.17 (1.15,1.19)**
2009	**1.14 (1.12,1.17)**	**1.14 (1.11,1.16)**	**1.16 (1.13,1.18)**	**1.19 (1.17,1.21)**
2010	**1.13 (1.10,1.16)**	**1.12 (1.10,1.15)**	**1.18 (1.16,1.20)**	**1.22 (1.20,1.24)**
2011	**1.16 (1.13,1.19)**	**1.16 (1.13,1.19)**	**1.20 (1.18,1.23)**	**1.25 (1.23,1.28)**
2012	**1.15 (1.13,1.18)**	**1.16 (1.13,1.19)**	**1.22 (1.19,1.24)**	**1.22 (1.19,1.24)**
2013	**1.17 (1.15,1.20)**	**1.18 (1.15,1.20)**	**1.27 (1.24,1.30)**	**1.26 (1.24,1.29)**
2014	**1.18 (1.15,1.21)**	**1.19 (1.16,1.22)**	**1.29 (1.26,1.32)**	**1.25 (1.23,1.28)**
2015	**1.20 (1.17,1.23)**	**1.20 (1.17,1.23)**	**1.28 (1.25,1.31)**	**1.26 (1.23,1.28)**
2016	**1.20 (1.17,1.23)**	**1.21 (1.18,1.24)**	**1.30 (1.27,1.33)**	**1.30 (1.27,1.33)**
2017	**1.23 (1.20,1.27)**	**1.23 (1.20,1.27)**	**1.30 (1.27,1.33)**	**1.31 (1.28,1.34)**
2018	**1.25 (1.21,1.29)**	**1.25 (1.21,1.29)**	**1.30 (1.27,1.33)**	**1.33 (1.30,1.36)**
2019	**1.29 (1.25,1.34)**	**1.29 (1.24,1.33)**	**1.31 (1.27,1.35)**	**1.35 (1.31,1.39)**
2020	**1.40 (1.35,1.46)**	**1.39 (1.34,1.45)**	**1.51 (1.46,1.56)**	**1.56 (1.51,1.62)**

*Note:* Data from the United States Surveillance, Epidemiology, and End Results Program 2000–2020. Model 1 adjusted for age. Model 2 adjusted for age, race/ethnicity, cancer site, and stage of the index cancer at diagnosis. Bold font indicates statistical significance at 0.05 level.

### 3.3 Trends by Cancer Characteristics

#### 3.3.1 Latency

**FIGURE 2:**
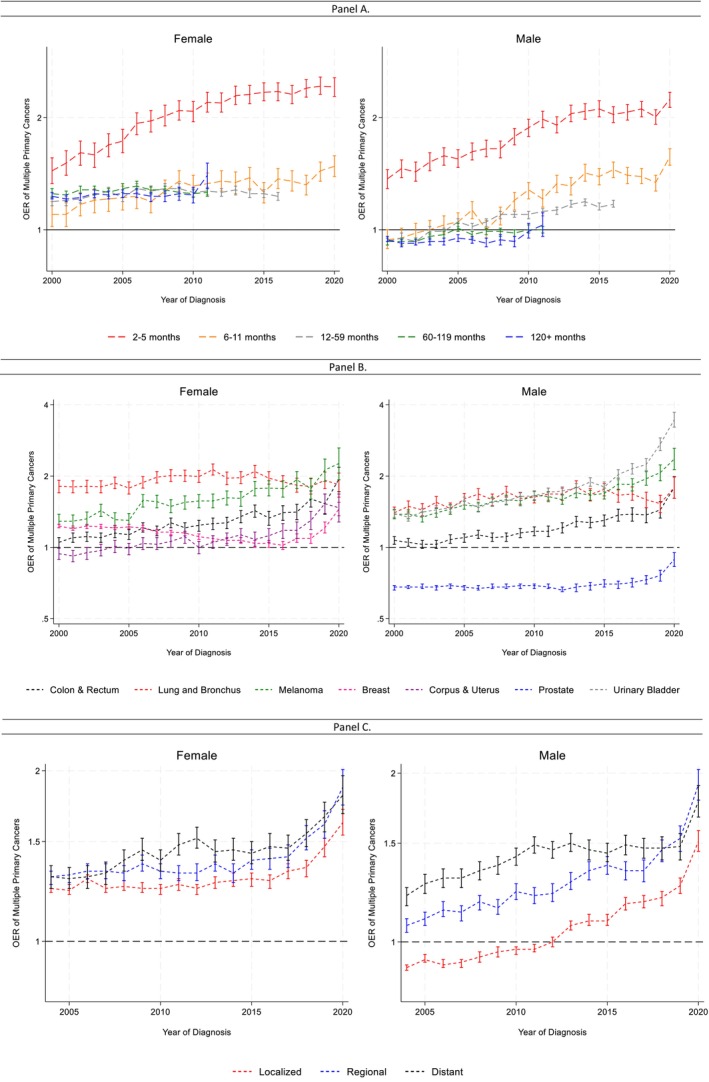
Observed/expected ratio (oer) of subsequent primary cancers by cancer characteristics among males and females over time. Data from the United States Surveilance, Epidemiology, and End Results Program 2000–2020. Brackets represent the 95% confidence interval of each estimate. Panel A: Latency. SPC risk increased over time across most latency periods for both males and females, with the highest OERs consistently observed for SPCs diagnosed within 12 months of the index cancer. Long‐latency SPCs (≥ 10 years) showed little change over time. Detailed Joinpoint results for each latency category are provided in Supporting Information. Panel B: Index cancer site. Temporal patterns varied by cancer site. Several common cancers (including colorectal, lung, melanoma, breast, prostate, and uterine cancers) showed rising SPC risk over the study period. Breast and lung cancers exhibited a decrease‐and‐increase pattern. Detailed Joinpoint results for each latency category are provided in Supporting Information. Full site‐ and latency‐stratified results are presented in Figure S1. Panel C: Stage of the index cancer. SPC risk increased over time across all stages, with survivors of distant‐stage cancers consistently experiencing the highest OERs. Increases were most pronounced in the later years of the study period. Detailed Joinpoint results for each latency category are provided in Supporting Information.

SPC risk increased over time across nearly all latency periods for both sexes (Figure 2A; Table S2). The highest OERs occurred for SPCs diagnosed within 6 months of the index cancer, and these short‐latency SPCs showed the steepest increases over time. Trends for SPCs diagnosed 1–10 years after the index cancer were more modest, and SPCs occurring ≥ 10 years after diagnosis showed little change over the study period. Detailed APC estimates by latency are provided in Supporting Information and Table S2.

#### 3.3.2 Cancer Site

SPC trends varied by index cancer site (Figure 2B; Supporting Information; Table S3). Several of the most common cancers (including colorectal, lung, melanoma, breast, prostate, and uterine cancers) showed rising SPC risk over the study period, with the sharpest increases concentrated in short‐latency SPCs (≤ 12 months). Breast cancer survivors exhibited distinct latency‐specific patterns, with increasing OERs for SPCs diagnosed within 6 months but declining OERs for SPCs diagnosed 1–10 years after the index cancer (Figure S2). Lung cancer survivors were the only group in which the highest SPC risk occurred more than 5 years after the index diagnosis, whereas survivors of all other cancer sites had the highest SPC risks within 6 months of diagnosis.

#### 3.3.3 Stage

SPC risk increased over time across all stages of the index cancer (Figure 2C; Supporting Information; Table S4). Survivors of distant‐stage cancers consistently had the highest SPC risk, followed by regional and localized cancers. Increases were most pronounced in the later years of the study period. Sensitivity analyses using international multiple primary rules yielded similar results.

### 3.4 Trends by Demographic Characteristics

**FIGURE 3:**
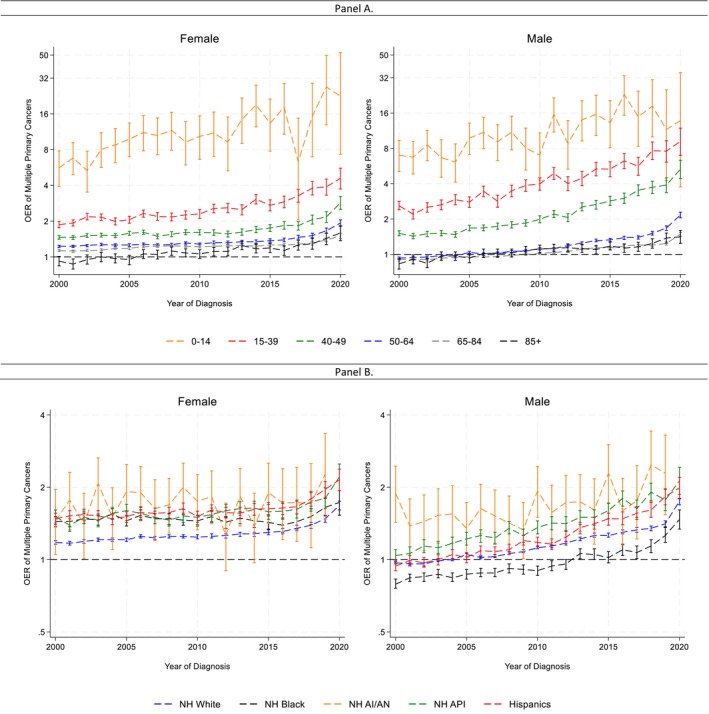
Observed/expected ratio (OER) of subsequent primary cancers by age and race/ethnicity among males and females over time. Data from the United States Surveillance, Epidemiology, and End Results Program 2000–2020. Brackets represent the 95% confidence interval of each estimate. Panel A: Age at diagnosis. SPC risk increased over time across all age groups for both males and females, with younger survivors consistently exhibiting the highest OERs. Patterns were broadly similar for males and females, although the magnitude and timing of increases varied by age. Detailed Joinpoint results for each age group are provided in Supporting Information. Panel B: Race/ethnicity. OERs increased over time across all racial/ethnic groups. American Indian/Alaska Native survivors had the highest OERs, though estimates were less precise due to smaller sample sizes. Temporal patterns differed modestly across groups, with more pronounced increases in recent years for several populations. Full Joinpoint results are presented in Supporting Information.

The risk of SPC increased over time across all age groups for both sexes (Figure 3 Panel A; Table S5). Notably, a clear age gradient was observed; younger survivors had higher OERs. OERs also increased across all racial/ethnic groups (Figure 3 Panel B; Table S6). Cancer survivors in the NH American Indian/Alaskan Native group had the highest OERs despite less precise estimates. Among males, NH Asian and Pacific Islander had the second highest OERs, while NH Black individuals had the lowest. Among females, NH White cancer survivors had the lowest OERs.

## 4 Discussion

To our knowledge, this is the first study to quantify contemporary trends in SPC risk across multiple cancer types and demographic groups using population‐based data. In this study, we observed a consistent increase in the relative risk of SPC among cancer survivors in the United States from 2000 to 2020. These findings support the integration of SPC risk into survivorship care planning and national cancer surveillance frameworks and provide key information for follow‐up guidelines.

Consistent with previous publications, patterns of SPC sites varied by index cancer [5, 31, 32]. Strong same‐site patterns observed for breast, lung, bladder, and melanoma survivors are consistent with shared etiologic pathways, persistent field cancerization, and ongoing exposure to underlying risk factors [5, 31, 32]. These findings reinforce the importance of site‐specific surveillance strategies, particularly for cancers with high multifocal potential. In contrast, colorectal, uterine, and prostate cancer survivors frequently developed SPCs in other common cancer sites (e.g., breast, lung, and bladder), highlighting the need for broad survivorship care that addresses shared behavioral and environmental risk factors. The concentration of SPCs in a limited number of sites across multiple index cancers highlights the importance of focused surveillance for lung and bronchus, colon and rectum, and melanoma, as well as breast in females and prostate and bladder in males. Together, these patterns provide actionable context for tailoring surveillance and prevention strategies beyond the overall elevated SPC risk.

The elevated risk of SPCs compared to the general population has become more pronounced over the past two decades [5]. This trend likely reflects a multifactorial interplay of systemic changes, including advances in diagnostic capabilities, changes in SPC coding rules that have simplified the differentiation between SPCs and metastases, and intensification of follow‐up protocols. The observed increases may also represent a true rise in SPC incidence driven by persistent environmental exposures (e.g., radon, viruses, and occupational hazards), lifestyle factors (e.g., smoking, obesity, alcohol use, and physical inactivity), and the long‐term effects of prior cancer treatments among survivors [33, 34, 35]. For example, the increases in SPC risk among colorectal and melanoma cancer survivors may coincide with improved imaging, expansion of screening guidelines, and advancements in treatment (such as immunotherapies and targeted therapies) introduced in the last decade [36, 37, 38], as well as persistent lifestyle factors such as diet and sun exposure. It is noteworthy that, as the population of cancer survivors grows, due in part to advances in systemic therapies that prolong survival and thus extend the period at risk for SPCs, even a stable incidence of SPCs translates to a greater absolute number of affected individuals.

Peak SPC incidence occurred within 6 months of the index diagnosis, likely reflecting diagnostic intensity and early detection. Early SPCs complicate treatment planning and may require coordinated, multidisciplinary care [39], including simultaneous management of two distinct cancers and potential therapeutic conflicts. Importantly, SPC risk remains elevated even after many years after the initial diagnosis, underscoring the need for long‐term surveillance and preventive care.

Although females consistently had higher relative risks of SPC, male cancer survivors had a faster increase over time. Male bladder cancer survivors, in particular, showed a steep rise in SPC risk, especially within 6–11 months of the index diagnosis. Given the strong association between smoking and bladder cancer [40], as well as many other cancers [12], this group may benefit from targeted follow‐up and smoking cessation interventions.

An exception to the overall pattern was observed among male prostate cancer survivors, who had significantly lower SPC risk compared to the general population [4, 5, 39, 41, 42]. The lower overall SPC OER among prostate cancer survivors likely reflects several factors. First, widespread PSA testing in the early 2000s led to overdiagnosis of indolent prostate tumors that may never have become clinically relevant during a patient's lifetime [43]; these survivors may have inherently low susceptibility to SPCs, thereby attenuating the overall OER. Second, prostate cancer is often managed with active surveillance or local therapy without systemic cytotoxic agents, limiting treatment‐related SPC risks [44]. Third, prostate cancers are usually diagnosed at an older age [44]. Older survivors may have substantial competing mortality. Previous studies found higher noncancer mortality among prostate cancer survivors compared to the general population [45], reducing the probability of living long enough to develop a SPC. Lifestyle differences may also contribute, as some studies documented behavioral changes after prostate cancer diagnosis [46, 47], although definitive evidence remains limited. Further research is needed to elucidate the mechanisms underlying this observation.

A novel finding was the U‐shaped trend in SPC risk among breast cancer survivors, driven by latency‐specific patterns. While short‐latency SPCs (within 6 months) increased over time, SPCs diagnosed 1–10 years after the index cancer declined. The reasons for these diverging trends are not immediately clear. The increase in short‐latency SPCs may reflect more intensive early surveillance, expanding screening practices, and advances in imaging (such as high‐resolution MRI and PET) that can uncover SPCs that might previously have gone undetected following the index diagnosis [48, 49, 50]. Expansion of screening guidelines may also contribute to greater detection and classification of additional primaries soon after initial cancer identification [50]. These factors can lead to earlier discovery of synchronous or closely timed malignancies that might otherwise have been detected later. The decline in SPCs diagnosed between 1 and 10 years post‐index cancer may result from reduced surveillance intensity over time, survivorship selection whereby patients with better prognosis remain at lower risk for new cancers, and improved therapies that lower long‐term SPC risk [49]. Together, these factors illustrate how evolving practices in screening, surveillance, and therapy can shape observed SPC patterns across different time intervals. Nonetheless, the risk of SPCs more than 5 years after the index cancer remained consistently elevated, supporting the need to extend surveillance intervals beyond 5 years, particularly given high survival rates and the rising incidence of breast cancer among younger women [3].

Lung cancer was the only cancer type where the highest SPC risk was observed at longer latencies (≥ 5 years), likely reflecting historically low survival rates [3], which had prevented lung cancer patients from surviving long enough to be at significant risk of developing SPCs. However, recent improvements in early detection, driven by expanded low‐dose CT screening guidelines, and advances in targeted therapies and immunotherapies have contributed to substantial gains in lung cancer survival: 5‐year survival increased from 15.5% in 2000 to 29.5% in 2017 [51]. These improvements have expanded the population of long‐term lung cancer survivors, the population at risk for developing SPCs. It is noteworthy that the risk of short‐latency SPCs doubled between 2000 and 2020, reaching levels comparable to long‐latency SPCs. This uptick in early SPC may be multifaceted, including more frequent use of comprehensive imaging, enhanced diagnostic sensitivity, and the adoption of routine surveillance for high‐risk patients [37]. Improvements in coding rules, which more clearly delineate subsequent primaries from metastatic disease or recurrences [20], may additionally contribute to the apparent rise in short‐latency SPCs. Nonetheless, as lung cancer survival improves, continued screening and smoking cessation efforts will be critical for this population.

The higher SPC risks among survivors of more advanced disease warrant careful interpretation. First, patients with advanced cancers are more likely to receive intensive multimodal therapy, including multi‐agent chemotherapy and prolonged exposure to cytotoxic agents, which are established risk factors for therapy‐related malignancies [10, 52]. Second, diagnostic complexity in metastatic disease raises the possibility of misclassifying metastatic progression as a new primary cancer. Although SEER registries apply standardized multiple primary rules and rigorous quality control procedures, some misclassification remains possible, particularly when histologic features overlap or when metastatic lesions undergo extensive diagnostic evaluation. Our exclusion of cancers diagnosed within 2 months of the index cancer helps mitigate short‐latency misclassification. Finally, metastatic survivors may represent a selected subgroup with distinct clinical trajectories, including prolonged treatment exposure and intensive surveillance, which may increase opportunities for SPC detection. Future studies are needed to clarify the mechanisms underlying these stage‐specific patterns.

We also observed a clear age gradient in SPC risk: younger cancer survivors had significantly higher relative risks compared to their peers in the general population. This is consistent with the role of inherited predispositions and the long‐term effects of early‐life cancer treatment [53, 54, 55]. Despite these differences, all age groups showed increasing trends over time, emphasizing the need for age‐sensitive follow‐up strategies. Genetic counseling may be particularly beneficial for younger survivors [54].

Disparities in SPC burden across racial and ethnic groups raise concerns about equitable access to survivorship care [56]. Studies have shown that American Indian/Alaska Native individuals are more likely to be diagnosed at advanced stages of cancer [57], which may necessitate more intensive therapeutic exposures and contribute to elevated risks of subsequent primary cancers (SPCs). In this context, the persistently high observed‐to‐expected ratios (OERs) among American Indian/Alaska Native survivors underscore the need for targeted interventions, including effective follow‐up strategies, culturally tailored lifestyle support, and improved access to genetic counseling [58]. Incorporating social determinants of health into survivorship models may help mitigate these disparities and inform more equitable cancer care.

A major strength of this study is the use of a large, population‐based cohort from the SEER Program, which provides comprehensive and high‐quality cancer data. Several limitations should be noted. First, SEER does not capture key risk factors such as genetic predisposition, screening and diagnostic procedures, lifestyle behaviors, environmental exposures, or treatment details, limiting our ability to explore these contributors. As a result, we could not disentangle the effect of surveillance intensity around the time of index cancer diagnosis from true biological increases. Future research with information about treatment, surveillance histories, and clinical outcomes is needed to shed light on potential mechanisms underlying the observed increasing trend of SPCs. Second, although SEER collects standardized sociodemographic and tumor characteristics, some variables may be subject to misclassification or incomplete reporting. Race/ethnicity is abstracted from medical records and may not fully capture multiracial identities or changes over time. With respect to SPCs, SEER does not track individuals across registries; thus, SPCs diagnosed after migration out of the registry catchment area are not captured, leading to potential under‐ascertainment of SPCs and inflation of follow‐up duration, which can attenuate SPC incidence estimates. In addition, SEER multiple primary coding rules changed in 2007 (and updated in 2018) [20, 21], which could have influenced the classification of some cancers as new primaries and thereby affected SPC estimates. Nonetheless, we did not observe any abrupt shifts in estimates between 2007 and 2008, suggesting that the impact of this transition on our results was limited. These sources of measurement error may introduce misclassification, although such misclassification is unlikely to explain the consistent temporal patterns observed across multiple subgroups. Third, despite the population‐based nature, SEER is not nationally representative. A recent comparison with the National Program of Cancer Registries (which covers nearly the entire US population) suggests similar age and sex distributions, but SEER disproportionately represents western regions [59]. Therefore, generalizability to the entire US population or to non‐US settings may be limited. Nonetheless, many of the underlying trends we observed, such as the growing survivor population and rising burden of early‐onset cancers, are consistent with global patterns [60, 61, 62]. Finally, because SEER 17 registries began in 2000, cancers diagnosed before 2000 may not be fully identifiable, potentially leading to misclassification of prior cancer history. This limitation does not affect the validity of our estimates for index cancers diagnosed from 2000 onward, which defines our analytic cohort, but it may underestimate the proportion of survivors with multiple prior cancers.

## 5 Conclusion

These findings underscore the urgent need for survivorship care models that incorporate personalized, risk‐based surveillance strategies tailored to latency patterns, cancer type, and sociodemographic factors. To address persistent disparities in SPC burden, future efforts should prioritize equitable access to long‐term follow‐up and preventive services—particularly among populations at highest risk. Identifying and addressing modifiable risk factors will be essential to reducing the growing burden of SPCs in the expanding population of cancer survivors.

## Author Contributions

**Hui G. Cheng:** conceptualization (equal), data curation (lead), formal analysis (lead), investigation (lead), methodology (lead), visualization (lead), writing – original draft (lead). **Livingstone Aduse‐Poku:** conceptualization (equal), methodology (supporting), validation (lead), writing – review and editing (equal). **Oxana Palesh:** conceptualization (equal), investigation (equal), methodology (equal), supervision (equal), writing – review and editing (equal). **Susan Hong:** conceptualization (equal), investigation (equal), methodology (equal), resources (equal), supervision (equal), writing – review and editing (equal).

## Funding

This work was supported by the US National Institute of Health (NCI P30CA016059; R01CA239714; R01CA172145, and R01CA226080). Funding agencies and sponsors had no role in the design and conduct of the study; collection, management, analysis, and interpretation of the data; preparation, review, or approval of the manuscript; and decision to submit the manuscript for publication.

## Conflicts of Interest

Dr. Palesh serves as a consultant for Brigham Young University, University of Rochester, Shook, Hardy and Bacon, Elsevier, Merck, NIH, Sage Publisher, and Monash University. Other authors declare no conflicts of interest relevant to this study.

## Supporting information


**Figure S1:** Observed/expected ratio (OER) of multiple primary cancers by site and latency of the index cancer among males over time. Data from the United States Surveillance, Epidemiology, and End Results Program 2000–2021.
**Figure S2:** Observed/expected ratio (OER) of multiple primary cancers by site and Latency of the Index Cancer among Females over Time. Data from the United States Surveillance, Epidemiology, and End Results Program 2000–2021.
**Figure S3:** Observed/expected ratio (OER) of Multiple Primary Cancers by Site of Index Cancer and Latency among Males and Females over Time. Data from the United States Surveillance, Epidemiology, and End Results Program 2000–2021.

**Table S1:** (1) Risk of subsequent primary cancer after index primary cancers in the 17 SEER registries from 2000 to 2021. (2) Risk of subsequent primary cancer (SPC) after index primary cancers excluding SPC with latency < 6 months. Data from the 17 SEER registries from 2000 to 2021.
**Table S2:** Risk of subsequent primary cancer after index primary cancers by latency in the 17 SEER registries from 2000 to 2021.
**Table S3:** Risk of subsequent primary cancer after index primary cancers by site of index cancer in the 17 SEER registries from 2000 to 2021.
**Table S4:** Risk of subsequent primary cancer after index primary cancers by stage of the index cancer in the 17 SEER registries from 2000 to 2021.
**Table S5:** Risk of subsequent primary cancer after index primary cancers by age at diagnosis of the index cancer in the 17 SEER registries from 2000 to 2021.
**Table S6:** Risk of subsequent primary cancer after index primary cancers by race/ethnicity in the 17 SEER registries from 2000 to 2021.

**Data S1:** Supporting Information.

## Data Availability

The data underlying this article are available through National Cancer Insititute Surveillance Epidemiology, End Results Program via SEER*Stat software https://seer.cancer.gov/help/seerstat.
