# Supplementary material for: Rising Risk of Subsequent Primary Cancers Among US Cancer Survivors, 2000–2021
Source: Cancer Med. 2026 Apr 6;15(4):e71778. doi: 10.1002/cam4.71778 (PMC13052203; doi:10.1002/cam4.71778)
Supplement: Supplementary file 1 — Figure S1: Observed/expected ratio (OER) of multiple primary cancers by site and latency of the index cancer among males over time. Data from the United States Surveillance, Epidemiology, and End Results Program 2000–2021. Figure S2: Observed/expected ratio (OER) of multiple primary cancers by site and Latency of the Index Cancer among Females over Time. Data from the United States Surveillance, Epidemiology, and End Results Program 2000–2021. Figure S3: Observed/expected ratio (OER) of Multiple Primary Cancers by Site of Index Cancer and Latency among Males and Females over Time. Data from the United States Surveillance, Epidemiology, and End Results Program 2000–2021. [file CAM4-15-e71778-s002.docx]

**Supplemental material**

| Figure S1. |
| --- |
| **Male prostate and uterine bladder cancers** |
| **** |
| Figure S1. Observed/Expected Ratio (OER) of Multiple Primary Cancers by Site and Latency of the Index Cancer among Males over Time. Data from the United States Surveillance, Epidemiology, and End Results Program 2000-2021.  Brackets represents the 95% confidece interval of each estimate. |

| Figure S2. |
| --- |
| **Female breast and uterine cancers** |
| **** |
| Figure S2. Observed/Expected Ratio (OER) of Multiple Primary Cancers by Site and Latency of the Index Cancer among Females over Time. Data from the United States Surveillance, Epidemiology, and End Results Program 2000-2021.  Brackets represents the 95% confidece interval of each estimate. |

| Figure S3. |
| --- |
| **Lung and bronchus** |
| **** |

| **Colorectal cancers** |
| --- |
| **** |
| **Melanoma** |
| **** |
| Figure S3. Observed/Expected Ratio (OER) of Multiple Primary Cancers by Site of Index Cancer and Latency among Males and Females over Time. Data from the United States Surveillance, Epidemiology, and End Results Program 2000-2021.  Brackets represents the 95% confidece interval of each estimate. |
