# Supplementary material for: Rising Risk of Subsequent Primary Cancers Among US Cancer Survivors, 2000–2021
Source: Cancer Med. 2026 Apr 6;15(4):e71778. doi: 10.1002/cam4.71778 (PMC13052203; doi:10.1002/cam4.71778)
Supplement: Supplementary file 2 — Table S1: (1) Risk of subsequent primary cancer after index primary cancers in the 17 SEER registries from 2000 to 2021. (2) Risk of subsequent primary cancer (SPC) after index primary cancers excluding SPC with latency < 6 months. Data from the 17 SEER registries from 2000 to 2021. Table S2: Risk of subsequent primary cancer after index primary cancers by latency in the 17 SEER registries from 2000 to 2021. Table S3: Risk of subsequent primary cancer after index primary cancers by site of index cancer in the 17 SEER registries from 2000 to 2021. Table S4: Risk of subsequent primary cancer after index primary cancers by stage of the index cancer in the 17 SEER registries from 2000 to 2021. Table S5: Risk of subsequent primary cancer after index primary cancers by age at diagnosis of the index cancer in the 17 SEER registries from 2000 to 2021. Table S6: Risk of subsequent primary cancer after index primary cancers by race/ethnicity in the 17 SEER registries from 2000 to 2021. [file CAM4-15-e71778-s003.docx]

| **Table S1-1. Risk of Subsequent Primary Cancer After Index Primary Cancers in the 17 SEER Registries From 2000 to 2021** | | | | | | | | | | | |
| --- | --- | --- | --- | --- | --- | --- | --- | --- | --- | --- | --- |
| Year of Index Primary Cancer | **Females** | | | | |  | **Males** | | | | |
|  | # of Index Primary Cancer | Person-Years of Follow Up | # of Subsequent Primary Cancer | | Observed/Expected Ratio  (95% CI) |  | # of Index Primary Cancer | Person-Years of Follow Up | # of Subsequent Primary Cancer | | Observed/Expected Ratio  (95% CI) |
|  |  |  | Observed | Expected |  |  |  |  | Observed | Expected |  |
| 2000 | 124110 | 1317714 | 20111 | 16389.0 | 1.23 (1.21, 1.24) |  | 132990 | 1269042 | 26226 | 27500.8 | 0.95 (0.94, 0.97) |
| 2001 | 128605 | 1341743 | 20215 | 16590.2 | 1.22 (1.20, 1.24) |  | 138151 | 1313329 | 26895 | 28169.6 | 0.95 (0.94, 0.97) |
| 2002 | 129022 | 1310313 | 20000 | 16071.3 | 1.24 (1.23, 1.26) |  | 139373 | 1312355 | 26555 | 27820.9 | 0.95 (0.94, 0.97) |
| 2003 | 127726 | 1254313 | 19112 | 15121.8 | 1.26 (1.25, 1.28) |  | 136708 | 1243637 | 25403 | 25751.2 | 0.99 (0.97, 1.00) |
| 2004 | 129996 | 1242464 | 18822 | 14829.2 | 1.27 (1.25, 1.29) |  | 140379 | 1251876 | 25286 | 25415.4 | 0.99 (0.98, 1.01) |
| 2005 | 131749 | 1224229 | 18429 | 14484.0 | 1.27 (1.25, 1.29) |  | 139699 | 1208393 | 24896 | 24046.0 | 1.04 (1.02, 1.05) |
| 2006 | 133427 | 1189450 | 18413 | 14055.9 | 1.31 (1.29, 1.33) |  | 145639 | 1235204 | 25008 | 24515.0 | 1.02 (1.01, 1.03) |
| 2007 | 136540 | 1167918 | 17649 | 13701.6 | 1.29 (1.27, 1.31) |  | 150993 | 1245628 | 25059 | 24372.8 | 1.03 (1.02, 1.04) |
| 2008 | 139720 | 1139698 | 17342 | 13268.1 | 1.31 (1.29, 1.33) |  | 150474 | 1175194 | 23656 | 22300.5 | 1.06 (1.05, 1.07) |
| 2009 | 142600 | 1105365 | 16800 | 12774.3 | 1.32 (1.30, 1.34) |  | 151861 | 1130095 | 22668 | 21007.5 | 1.08 (1.07, 1.09) |
| 2010 | 142062 | 1033977 | 15457 | 11901.3 | 1.30 (1.28, 1.32) |  | 151338 | 1051723 | 21458 | 19269.6 | 1.11 (1.10, 1.13) |
| 2011 | 143970 | 980610 | 14825 | 11196.4 | 1.32 (1.30, 1.35) |  | 152161 | 991851 | 20296 | 17969.5 | 1.13 (1.11, 1.15) |
| 2012 | 147149 | 926184 | 13878 | 10510.0 | 1.32 (1.30, 1.34) |  | 147727 | 873576 | 17898 | 15415.8 | 1.16 (1.14, 1.18) |
| 2013 | 148072 | 850655 | 12913 | 9612.7 | 1.34 (1.32, 1.37) |  | 147739 | 799335 | 17057 | 13958.7 | 1.22 (1.20, 1.24) |
| 2014 | 151925 | 790785 | 11884 | 8844.8 | 1.34 (1.32, 1.37) |  | 148715 | 723759 | 15567 | 12337.6 | 1.26 (1.24, 1.28) |
| 2015 | 156057 | 720143 | 10869 | 7980.6 | 1.36 (1.34, 1.39) |  | 152975 | 667268 | 14340 | 11322.2 | 1.27 (1.25, 1.29) |
| 2016 | 157556 | 627039 | 9526 | 6956.4 | 1.37 (1.34, 1.40) |  | 156037 | 593070 | 13131 | 10007.9 | 1.31 (1.29, 1.33) |
| 2017 | 158394 | 526893 | 8162 | 5794.6 | 1.41 (1.38, 1.44) |  | 159724 | 511909 | 11413 | 8593.3 | 1.33 (1.30, 1.35) |
| 2018 | 158656 | 420195 | 6694 | 4581.4 | 1.46 (1.43, 1.50) |  | 159539 | 409131 | 9296 | 6778.4 | 1.37 (1.34, 1.40) |
| 2019 | 162042 | 311010 | 5212 | 3325.5 | 1.57 (1.53, 1.61) |  | 165005 | 309255 | 7254 | 5034.8 | 1.44 (1.41, 1.47) |
| 2020 | 145855 | 164979 | 3044 | 1726.4 | 1.76 (1.70, 1.83) |  | 148059 | 164886 | 4608 | 2632.4 | 1.75 (1.70, 1.80) |

| **Table S1-2. Risk of Subsequent Primary Cancer (SPC) After Index Primary Cancers Excluding SPC with Latency<6 Months. Data from the 17 SEER Registries From 2000 to 2021** | | | | | | | | | | | |
| --- | --- | --- | --- | --- | --- | --- | --- | --- | --- | --- | --- |
| Year of Index Primary Cancer | **Females** | | | | |  | **Males** | | | | |
|  | # of Index Primary Cancer | Person-Years of Follow Up | # of Subsequent Primary Cancer | | Observed/Expected Ratio  (95% CI) |  | # of Index Primary Cancer | Person-Years of Follow Up | # of Subsequent Primary Cancer | | Observed/Expected Ratio  (95% CI) |
|  |  |  | Observed | Expected |  |  |  |  | Observed | Expected |  |
| 2000 | 113,126 | 1,284,816 | 19,477 | 15,940 | 1.22 (1.20, 1.24) |  | 119,705 | 1,235,863 | 25,101 | 26,812 | 0.94 (0.92, 0.95) |
| 2001 | 117,223 | 1,308,631 | 19,538 | 16,130 | 1.21 (1.19, 1.23) |  | 124,629 | 1,280,397 | 25,661 | 27,493 | 0.93 (0.92, 0.94) |
| 2002 | 117,805 | 1,276,327 | 19,270 | 15,616 | 1.23 (1.22, 1.25) |  | 126,317 | 1,279,580 | 25,336 | 27,179 | 0.93 (0.92, 0.94) |
| 2003 | 116,493 | 1,221,257 | 18,405 | 14,699 | 1.25 (1.23, 1.27) |  | 123,593 | 1,210,729 | 24,134 | 25,123 | 0.96 (0.95, 0.97) |
| 2004 | 118,826 | 1,209,045 | 18,065 | 14,413 | 1.25 (1.24, 1.27) |  | 127,499 | 1,218,658 | 23,963 | 24,838 | 0.96 (0.95, 0.98) |
| 2005 | 120,579 | 1,189,366 | 17,639 | 14,073 | 1.25 (1.23, 1.27) |  | 126,917 | 1,175,468 | 23,633 | 23,508 | 1.01 (0.99, 1.02) |
| 2006 | 122,200 | 1,156,333 | 17,526 | 13,652 | 1.28 (1.26, 1.30) |  | 132,927 | 1,202,382 | 23,640 | 23,956 | 0.99 (0.97, 1.00) |
| 2007 | 125,294 | 1,133,877 | 16,732 | 13,299 | 1.26 (1.24, 1.28) |  | 138,368 | 1,212,639 | 23,608 | 23,822 | 0.99 (0.98, 1.00) |
| 2008 | 128,716 | 1,105,046 | 16,373 | 12,874 | 1.27 (1.25, 1.29) |  | 138,161 | 1,142,582 | 22,236 | 21,789 | 1.02 (1.01, 1.03) |
| 2009 | 131,474 | 1,068,926 | 15,778 | 12,363 | 1.28 (1.26, 1.30) |  | 139,721 | 1,098,138 | 21,160 | 20,554 | 1.03 (1.02, 1.04) |
| 2010 | 131,351 | 997,652 | 14,452 | 11,504 | 1.26 (1.24, 1.28) |  | 139,157 | 1,021,966 | 19,983 | 18,915 | 1.06 (1.04, 1.07) |
| 2011 | 133,518 | 942,595 | 13,750 | 10,790 | 1.27 (1.25, 1.30) |  | 140,576 | 959,218 | 18,729 | 17,558 | 1.07 (1.05, 1.08) |
| 2012 | 136,477 | 887,552 | 12,778 | 10,103 | 1.26 (1.24, 1.29) |  | 135,677 | 841,577 | 16,439 | 15,014 | 1.09 (1.08, 1.11) |
| 2013 | 137,461 | 811,705 | 11,752 | 9,207 | 1.28 (1.25, 1.30) |  | 135,824 | 766,783 | 15,475 | 13,533 | 1.14 (1.13, 1.16) |
| 2014 | 141,389 | 749,981 | 10,685 | 8,426 | 1.27 (1.24, 1.29) |  | 136,987 | 689,842 | 14,000 | 11,893 | 1.18 (1.16, 1.20) |
| 2015 | 145,511 | 677,923 | 9,615 | 7,550 | 1.27 (1.25, 1.30) |  | 141,366 | 632,124 | 12,718 | 10,868 | 1.17 (1.15, 1.19) |
| 2016 | 147,225 | 583,655 | 8,241 | 6,508 | 1.27 (1.24, 1.29) |  | 144,818 | 556,471 | 11,514 | 9,524 | 1.21 (1.19, 1.23) |
| 2017 | 147,796 | 482,430 | 6,902 | 5,329 | 1.30 (1.26, 1.33) |  | 148,793 | 473,216 | 9,705 | 8,060 | 1.20 (1.18, 1.23) |
| 2018 | 148,742 | 374,845 | 5,364 | 4,102 | 1.31 (1.27, 1.34) |  | 149,212 | 367,961 | 7,532 | 6,176 | 1.22 (1.19, 1.25) |
| 2019 | 151,749 | 263,012 | 3,841 | 2,821 | 1.36 (1.32, 1.41) |  | 154,407 | 264,149 | 5,519 | 4,361 | 1.27 (1.23, 1.30) |
| 2020 | 135,484 | 121,090 | 1,880 | 1,277 | 1.47 (1.41, 1.54) |  | 137,335 | 122,480 | 2,949 | 1,998 | 1.48 (1.42, 1.53) |

| **Table S2. Risk of Subsequent Primary Cancer After Index Primary Cancers by Latency in the 17 SEER Registries From 2000 to 2021** | | | | | | | | | | | | | | |  |  |
| --- | --- | --- | --- | --- | --- | --- | --- | --- | --- | --- | --- | --- | --- | --- | --- | --- |
| Year of Index Primary Cancer | **Females** | | | | | |  | | **Males** | | | | | | |  |
|  | Latency | # of Index Primary Cancer | Person-Years of Follow Up | # of Subsequent Primary Cancer | | Observed/  Expected Ratio  (95% CI) | |  | | # of Index Primary Cancer | Person-Years of Follow Up | # of Subsequent Primary Cancer | | Observed/  Expected Ratio  (95% CI) | | |
|  |  |  |  | Observed | Expected |  |  |  | |  |  | Observed | Expected |  |  |  |
| 2000 | 2-5 months | 124480 | 38869 | 635 | 441.2 | 1.44 (1.33, 1.56) | |  | | 133536 | 41339 | 1133 | 826.2 | 1.37 (1.29, 1.45) | | |
| 2001 |  | 129062 | 40295 | 689 | 455.8 | 1.51 (1.40, 1.63) | |  | | 138810 | 43026 | 1246 | 855.9 | 1.46 (1.38, 1.54) | | |
| 2002 |  | 129439 | 40448 | 733 | 456.3 | 1.61 (1.49, 1.73) | |  | | 140103 | 43526 | 1232 | 859.6 | 1.43 (1.35, 1.52) | | |
| 2003 |  | 128194 | 40112 | 713 | 448.7 | 1.59 (1.47, 1.71) | |  | | 137385 | 42738 | 1276 | 837.1 | 1.52 (1.44, 1.61) | | |
| 2004 |  | 130511 | 40800 | 766 | 454.4 | 1.69 (1.57, 1.81) | |  | | 141144 | 43898 | 1335 | 846.8 | 1.58 (1.49, 1.66) | | |
| 2005 |  | 132250 | 41378 | 792 | 457.7 | 1.73 (1.61, 1.86) | |  | | 140492 | 43689 | 1265 | 816.1 | 1.55 (1.47, 1.64) | | |
| 2006 |  | 134093 | 41914 | 895 | 463.9 | 1.93 (1.80, 2.06) | |  | | 146640 | 45730 | 1385 | 854.2 | 1.62 (1.54, 1.71) | | |
| 2007 |  | 137261 | 43076 | 930 | 475.0 | 1.96 (1.83, 2.09) | |  | | 152202 | 47685 | 1466 | 888.7 | 1.65 (1.57, 1.74) | | |
| 2008 |  | 140553 | 44074 | 983 | 485.5 | 2.02 (1.90, 2.16) | |  | | 151756 | 47455 | 1441 | 875.3 | 1.65 (1.56, 1.73) | | |
| 2009 |  | 143413 | 45009 | 1033 | 493.5 | 2.09 (1.97, 2.22) | |  | | 153364 | 47951 | 1525 | 856.0 | 1.78 (1.69, 1.87) | | |
| 2010 |  | 142942 | 44930 | 1015 | 489.0 | 2.08 (1.95, 2.21) | |  | | 153141 | 47805 | 1501 | 799.0 | 1.88 (1.78, 1.98) | | |
| 2011 |  | 144822 | 45768 | 1093 | 496.2 | 2.20 (2.07, 2.34) | |  | | 153887 | 48332 | 1598 | 807.6 | 1.98 (1.88, 2.08) | | |
| 2012 |  | 148127 | 46680 | 1113 | 507.1 | 2.19 (2.07, 2.33) | |  | | 149493 | 46628 | 1488 | 777.8 | 1.91 (1.82, 2.01) | | |
| 2013 |  | 149173 | 47005 | 1176 | 512.4 | 2.29 (2.17, 2.43) | |  | | 149605 | 46673 | 1607 | 782.8 | 2.05 (1.95, 2.16) | | |
| 2014 |  | 153078 | 48309 | 1210 | 524.8 | 2.31 (2.18, 2.44) | |  | | 150715 | 47079 | 1614 | 777.0 | 2.08 (1.98, 2.18) | | |
| 2015 |  | 157393 | 49824 | 1270 | 541.9 | 2.34 (2.22, 2.48) | |  | | 155350 | 48670 | 1664 | 787.5 | 2.11 (2.01, 2.22) | | |
| 2016 |  | 159031 | 50262 | 1298 | 552.7 | 2.35 (2.22, 2.48) | |  | | 158773 | 49685 | 1652 | 810.6 | 2.04 (1.94, 2.14) | | |
| 2017 |  | 160000 | 50501 | 1286 | 557.6 | 2.31 (2.18, 2.44) | |  | | 162886 | 51052 | 1741 | 842.6 | 2.07 (1.97, 2.17) | | |
| 2018 |  | 160479 | 50753 | 1353 | 564.1 | 2.40 (2.27, 2.53) | |  | | 162839 | 51126 | 1794 | 851.1 | 2.11 (2.01, 2.21) | | |
| 2019 |  | 163966 | 51964 | 1387 | 569.8 | 2.43 (2.31, 2.57) | |  | | 168731 | 53080 | 1767 | 880.0 | 2.01 (1.92, 2.10) | | |
| 2020 |  | 147963 | 46387 | 1179 | 487.7 | 2.42 (2.28, 2.56) | |  | | 152046 | 47244 | 1694 | 757.9 | 2.24 (2.13, 2.34) | | |
| 2000 | 6-11 months | 113126 | 53559 | 662 | 600.5 | 1.10 (1.02, 1.19) | |  | | 119705 | 56222 | 1065 | 1128.7 | 0.94 (0.89, 1.00) | | |
| 2001 |  | 117223 | 55554 | 682 | 620.6 | 1.10 (1.02, 1.18) | |  | | 124629 | 58609 | 1117 | 1171.9 | 0.95 (0.90, 1.01) | | |
| 2002 |  | 117805 | 55814 | 729 | 621.9 | 1.17 (1.09, 1.26) | |  | | 126317 | 59453 | 1161 | 1180.4 | 0.98 (0.93, 1.04) | | |
| 2003 |  | 116493 | 55389 | 733 | 612.0 | 1.20 (1.11, 1.29) | |  | | 123593 | 58338 | 1150 | 1147.5 | 1.00 (0.95, 1.06) | | |
| 2004 |  | 118826 | 56450 | 748 | 620.4 | 1.21 (1.12, 1.30) | |  | | 127499 | 60099 | 1187 | 1147.7 | 1.03 (0.98, 1.09) | | |
| 2005 |  | 120579 | 57318 | 761 | 625.7 | 1.22 (1.13, 1.31) | |  | | 126917 | 59852 | 1180 | 1122.4 | 1.05 (0.99, 1.11) | | |
| 2006 |  | 122200 | 58181 | 780 | 636.6 | 1.23 (1.14, 1.31) | |  | | 132927 | 62883 | 1336 | 1181.4 | 1.13 (1.07, 1.19) | | |
| 2007 |  | 125294 | 59822 | 778 | 652.2 | 1.19 (1.11, 1.28) | |  | | 138368 | 65715 | 1234 | 1231.4 | 1.00 (0.95, 1.06) | | |
| 2008 |  | 128716 | 61431 | 852 | 669.5 | 1.27 (1.19, 1.36) | |  | | 138161 | 65502 | 1321 | 1213.9 | 1.09 (1.03, 1.15) | | |
| 2009 |  | 131474 | 62800 | 919 | 678.8 | 1.35 (1.27, 1.44) | |  | | 139721 | 66275 | 1364 | 1139.2 | 1.20 (1.13, 1.26) | | |
| 2010 |  | 131351 | 62733 | 888 | 676.5 | 1.31 (1.23, 1.40) | |  | | 139157 | 66010 | 1413 | 1107.8 | 1.28 (1.21, 1.34) | | |
| 2011 |  | 133518 | 64000 | 914 | 688.6 | 1.33 (1.24, 1.42) | |  | | 140576 | 66887 | 1365 | 1124.2 | 1.21 (1.15, 1.28) | | |
| 2012 |  | 136477 | 65246 | 951 | 703.8 | 1.35 (1.27, 1.44) | |  | | 135677 | 64197 | 1431 | 1074.6 | 1.33 (1.26, 1.40) | | |
| 2013 |  | 137461 | 65696 | 952 | 711.6 | 1.34 (1.25, 1.43) | |  | | 135824 | 64383 | 1420 | 1084.5 | 1.31 (1.24, 1.38) | | |
| 2014 |  | 141389 | 67674 | 1009 | 731.8 | 1.38 (1.29, 1.47) | |  | | 136987 | 64805 | 1501 | 1053.8 | 1.42 (1.35, 1.50) | | |
| 2015 |  | 145511 | 69887 | 955 | 756.8 | 1.26 (1.18, 1.34) | |  | | 141366 | 67205 | 1521 | 1092.5 | 1.39 (1.32, 1.46) | | |
| 2016 |  | 147225 | 70339 | 1057 | 771.3 | 1.37 (1.29, 1.46) | |  | | 144818 | 68678 | 1635 | 1127.7 | 1.45 (1.38, 1.52) | | |
| 2017 |  | 147796 | 70694 | 1054 | 778.9 | 1.35 (1.27, 1.44) | |  | | 148793 | 70632 | 1645 | 1174.3 | 1.40 (1.33, 1.47) | | |
| 2018 |  | 148742 | 71316 | 1044 | 791.0 | 1.32 (1.24, 1.40) | |  | | 149212 | 70919 | 1657 | 1188.9 | 1.39 (1.33, 1.46) | | |
| 2019 |  | 151749 | 72613 | 1115 | 776.9 | 1.44 (1.35, 1.52) | |  | | 154407 | 73513 | 1604 | 1199.1 | 1.34 (1.27, 1.40) | | |
| 2020 |  | 135484 | 64032 | 997 | 672.5 | 1.48 (1.39, 1.58) | |  | | 137335 | 64550 | 1637 | 1044.2 | 1.57 (1.49, 1.65) | | |
| 2000 | 12-59 months | 103537 | 345112 | 4683 | 3928.9 | 1.19 (1.16, 1.23) | |  | | 108145 | 355377 | 6957 | 7489.6 | 0.93 (0.91, 0.95) | | |
| 2001 |  | 107540 | 360173 | 4890 | 4087.8 | 1.20 (1.16, 1.23) | |  | | 112924 | 373800 | 7433 | 7792.3 | 0.95 (0.93, 0.98) | | |
| 2002 |  | 108073 | 362585 | 4981 | 4102.8 | 1.21 (1.18, 1.25) | |  | | 114557 | 381011 | 7305 | 7828.8 | 0.93 (0.91, 0.95) | | |
| 2003 |  | 106917 | 359210 | 5003 | 4024.6 | 1.24 (1.21, 1.28) | |  | | 112218 | 372077 | 7441 | 7494.3 | 0.99 (0.97, 1.02) | | |
| 2004 |  | 109410 | 368304 | 5204 | 4105.9 | 1.27 (1.23, 1.30) | |  | | 115980 | 385840 | 7612 | 7695.1 | 0.99 (0.97, 1.01) | | |
| 2005 |  | 111157 | 375661 | 5101 | 4166.0 | 1.22 (1.19, 1.26) | |  | | 115479 | 385462 | 7901 | 7550.6 | 1.05 (1.02, 1.07) | | |
| 2006 |  | 112970 | 382651 | 5481 | 4246.5 | 1.29 (1.26, 1.33) | |  | | 121574 | 408381 | 8017 | 7857.8 | 1.02 (1.00, 1.04) | | |
| 2007 |  | 115905 | 394064 | 5523 | 4354.1 | 1.27 (1.24, 1.30) | |  | | 126914 | 429325 | 8425 | 8055.6 | 1.05 (1.02, 1.07) | | |
| 2008 |  | 119412 | 405865 | 5783 | 4472.9 | 1.29 (1.26, 1.33) | |  | | 126761 | 426307 | 8435 | 7694.8 | 1.10 (1.07, 1.12) | | |
| 2009 |  | 122224 | 416319 | 5940 | 4587.6 | 1.29 (1.26, 1.33) | |  | | 128313 | 432071 | 8445 | 7684.0 | 1.10 (1.08, 1.12) | | |
| 2010 |  | 122012 | 415868 | 5794 | 4602.7 | 1.26 (1.23, 1.29) | |  | | 127808 | 430256 | 8388 | 7648.6 | 1.10 (1.07, 1.12) | | |
| 2011 |  | 124207 | 424132 | 5966 | 4698.1 | 1.27 (1.24, 1.30) | |  | | 129192 | 433803 | 8546 | 7649.4 | 1.12 (1.09, 1.14) | | |
| 2012 |  | 126951 | 433269 | 6048 | 4811.0 | 1.26 (1.23, 1.29) | |  | | 124123 | 414162 | 8082 | 7163.6 | 1.13 (1.10, 1.15) | | |
| 2013 |  | 127794 | 436272 | 6220 | 4874.9 | 1.28 (1.24, 1.31) | |  | | 124545 | 414267 | 8375 | 7133.8 | 1.17 (1.15, 1.20) | | |
| 2014 |  | 131763 | 450025 | 6271 | 5030.8 | 1.25 (1.22, 1.28) | |  | | 125262 | 415515 | 8425 | 7085.7 | 1.19 (1.16, 1.21) | | |
| 2015 |  | 135699 | 462796 | 6434 | 5157.7 | 1.25 (1.22, 1.28) | |  | | 129645 | 431795 | 8554 | 7414.3 | 1.15 (1.13, 1.18) | | |
| 2016 |  | 136884 | 463840 | 6367 | 5176.8 | 1.23 (1.20, 1.26) | |  | | 132932 | 441401 | 8894 | 7572.8 | 1.17 (1.15, 1.20) | | |
| 2000 | 60-119 months | 75931 | 338047 | 5142 | 4104.0 | 1.25 (1.22, 1.29) | |  | | 77315 | 337051 | 6969 | 7481.1 | 0.93 (0.91, 0.95) | | |
| 2001 |  | 79509 | 355692 | 5321 | 4306.1 | 1.24 (1.20, 1.27) | |  | | 81763 | 357925 | 7276 | 7805.7 | 0.93 (0.91, 0.95) | | |
| 2002 |  | 80082 | 358200 | 5514 | 4312.0 | 1.28 (1.25, 1.31) | |  | | 83703 | 367583 | 7336 | 7860.3 | 0.93 (0.91, 0.95) | | |
| 2003 |  | 79397 | 355008 | 5389 | 4220.6 | 1.28 (1.24, 1.31) | |  | | 81616 | 358051 | 7140 | 7429.5 | 0.96 (0.94, 0.98) | | |
| 2004 |  | 81536 | 365245 | 5436 | 4316.3 | 1.26 (1.23, 1.29) | |  | | 84818 | 372681 | 7316 | 7541.4 | 0.97 (0.95, 0.99) | | |
| 2005 |  | 83346 | 374890 | 5722 | 4424.2 | 1.29 (1.26, 1.33) | |  | | 84843 | 372746 | 7480 | 7391.7 | 1.01 (0.99, 1.04) | | |
| 2006 |  | 85044 | 381503 | 5952 | 4526.8 | 1.31 (1.28, 1.35) | |  | | 90259 | 398691 | 7724 | 7940.8 | 0.97 (0.95, 0.99) | | |
| 2007 |  | 87704 | 393201 | 5995 | 4677.7 | 1.28 (1.25, 1.31) | |  | | 95288 | 421274 | 8250 | 8348.4 | 0.99 (0.97, 1.01) | | |
| 2008 |  | 90221 | 404153 | 6163 | 4815.8 | 1.28 (1.25, 1.31) | |  | | 94251 | 416823 | 7982 | 8103.3 | 0.99 (0.96, 1.01) | | |
| 2009 |  | 92888 | 415716 | 6258 | 4966.1 | 1.26 (1.23, 1.29) | |  | | 95877 | 425177 | 8078 | 8219.2 | 0.98 (0.96, 1.00) | | |
| 2010 |  | 92542 | 413447 | 6185 | 4946.5 | 1.25 (1.22, 1.28) | |  | | 95024 | 420598 | 8118 | 8083.4 | 1.00 (0.98, 1.03) | | |
| 2011 |  | 94255 | 418352 | 6262 | 4968.5 | 1.26 (1.23, 1.29) | |  | | 95808 | 421919 | 8072 | 8060.2 | 1.00 (0.98, 1.02) | | |
| 2000 | 120+ months | 60842 | 548098 | 8990 | 7306.8 | 1.23 (1.21, 1.26) | |  | | 58858 | 487213 | 10110 | 10712.2 | 0.94 (0.93, 0.96) | | |
| 2001 |  | 64093 | 537212 | 8645 | 7115.9 | 1.21 (1.19, 1.24) | |  | | 62684 | 490062 | 9835 | 10723.3 | 0.92 (0.90, 0.94) | | |
| 2002 |  | 64464 | 499728 | 8046 | 6579.2 | 1.22 (1.20, 1.25) | |  | | 64676 | 471533 | 9534 | 10309.7 | 0.92 (0.91, 0.94) | | |
| 2003 |  | 63813 | 451651 | 7280 | 5841.7 | 1.25 (1.22, 1.28) | |  | | 62825 | 422262 | 8403 | 9051.8 | 0.93 (0.91, 0.95) | | |
| 2004 |  | 65818 | 419047 | 6677 | 5370.8 | 1.24 (1.21, 1.27) | |  | | 65507 | 400038 | 7848 | 8454.2 | 0.93 (0.91, 0.95) | | |
| 2005 |  | 67704 | 381496 | 6055 | 4857.4 | 1.25 (1.22, 1.28) | |  | | 65676 | 357408 | 7072 | 7443.0 | 0.95 (0.93, 0.97) | | |
| 2006 |  | 68724 | 333998 | 5313 | 4242.2 | 1.25 (1.22, 1.29) | |  | | 70395 | 332427 | 6563 | 6975.6 | 0.94 (0.92, 0.96) | | |
| 2007 |  | 70649 | 286790 | 4436 | 3615.4 | 1.23 (1.19, 1.26) | |  | | 74426 | 296325 | 5699 | 6186.4 | 0.92 (0.90, 0.95) | | |
| 2008 |  | 72725 | 233597 | 3575 | 2915.7 | 1.23 (1.19, 1.27) | |  | | 73680 | 233951 | 4498 | 4776.8 | 0.94 (0.91, 0.97) | | |
| 2009 |  | 74499 | 174091 | 2661 | 2131.0 | 1.25 (1.20, 1.30) | |  | | 75202 | 174615 | 3273 | 3511.9 | 0.93 (0.90, 0.96) | | |
| 2010 |  | 73856 | 105604 | 1585 | 1278.0 | 1.24 (1.18, 1.30) | |  | | 74017 | 105103 | 2064 | 2074.8 | 0.99 (0.95, 1.04) | | |
| 2011 |  | 73354 | 36110 | 608 | 435.2 | 1.40 (1.29, 1.51) | |  | | 72952 | 36609 | 746 | 723.8 | 1.03 (0.96, 1.11) | | |

| **Table S3. Risk of Subsequent Primary Cancer After Index Primary Cancers by Site of Index Cancer in the 17 SEER Registries From 2000 to 2021** | | | | | | |
| --- | --- | --- | --- | --- | --- | --- |
| Year of Index Primary Cancer | Site of Index Primary Cancer | # of Index Primary Cancer | Person-Years of Follow Up | # of Subsequent Primary Cancer | | Observed/Expected Ratio  (95% CI) |
|  |  |  |  | Observed | Expected |  |
|  | **Females** | | | | | |
| 2000 | Colorectal | 13729 | 122793 | 1974 | 1883.09 | 1.05 (1.00, 1.10) |
| 2001 |  | 13757 | 121537 | 2011 | 1832.47 | 1.10 (1.05, 1.15) |
| 2002 |  | 13800 | 121348 | 1996 | 1804.16 | 1.11 (1.06, 1.16) |
| 2003 |  | 13568 | 116543 | 1903 | 1723.68 | 1.10 (1.05, 1.15) |
| 2004 |  | 13292 | 113659 | 1892 | 1641.78 | 1.15 (1.10, 1.21) |
| 2005 |  | 13247 | 112036 | 1813 | 1600.98 | 1.13 (1.08, 1.19) |
| 2006 |  | 13292 | 107411 | 1838 | 1519.29 | 1.21 (1.16, 1.27) |
| 2007 |  | 13202 | 103308 | 1689 | 1437.71 | 1.17 (1.12, 1.23) |
| 2008 |  | 13167 | 98802 | 1731 | 1363.81 | 1.27 (1.21, 1.33) |
| 2009 |  | 13040 | 93487 | 1523 | 1261.25 | 1.21 (1.15, 1.27) |
| 2010 |  | 12740 | 86510 | 1404 | 1135.1 | 1.24 (1.17, 1.30) |
| 2011 |  | 12679 | 79412 | 1320 | 1045.88 | 1.26 (1.19, 1.33) |
| 2012 |  | 12675 | 75145 | 1231 | 967.89 | 1.27 (1.20, 1.34) |
| 2013 |  | 12258 | 66082 | 1138 | 841.77 | 1.35 (1.27, 1.43) |
| 2014 |  | 12787 | 63255 | 1117 | 783.43 | 1.43 (1.34, 1.51) |
| 2015 |  | 12745 | 55985 | 896 | 675.2 | 1.33 (1.24, 1.42) |
| 2016 |  | 13085 | 50445 | 842 | 602.4 | 1.40 (1.30, 1.50) |
| 2017 |  | 12783 | 41031 | 684 | 484.31 | 1.41 (1.31, 1.52) |
| 2018 |  | 12718 | 32882 | 615 | 384.55 | 1.60 (1.48, 1.73) |
| 2019 |  | 12813 | 24089 | 416 | 272.57 | 1.53 (1.38, 1.68) |
| 2020 |  | 11586 | 12935 | 275 | 141.89 | 1.94 (1.72, 2.18) |
| 2000 | Lung and Bronchus | 13254 | 40848 | 1065 | 589.79 | 1.81 (1.70, 1.92) |
| 2001 |  | 13507 | 41409 | 1094 | 606.17 | 1.80 (1.70, 1.91) |
| 2002 |  | 13557 | 40603 | 1075 | 594.66 | 1.81 (1.70, 1.92) |
| 2003 |  | 13695 | 41036 | 1074 | 595.44 | 1.80 (1.70, 1.91) |
| 2004 |  | 13665 | 42079 | 1155 | 616.91 | 1.87 (1.77, 1.98) |
| 2005 |  | 14009 | 44363 | 1149 | 645.77 | 1.78 (1.68, 1.89) |
| 2006 |  | 14203 | 44920 | 1222 | 648.95 | 1.88 (1.78, 1.99) |
| 2007 |  | 14201 | 44392 | 1275 | 643.32 | 1.98 (1.87, 2.09) |
| 2008 |  | 14218 | 43808 | 1273 | 632.11 | 2.01 (1.90, 2.13) |
| 2009 |  | 14506 | 43991 | 1269 | 631.89 | 2.01 (1.90, 2.12) |
| 2010 |  | 14072 | 42299 | 1213 | 608.85 | 1.99 (1.88, 2.11) |
| 2011 |  | 13978 | 41569 | 1271 | 596.32 | 2.13 (2.02, 2.25) |
| 2012 |  | 14185 | 39935 | 1114 | 569.29 | 1.96 (1.84, 2.08) |
| 2013 |  | 14302 | 39936 | 1130 | 572.48 | 1.97 (1.86, 2.09) |
| 2014 |  | 14385 | 38071 | 1134 | 541.72 | 2.09 (1.97, 2.22) |
| 2015 |  | 14587 | 37457 | 1042 | 530.76 | 1.96 (1.85, 2.09) |
| 2016 |  | 14771 | 35566 | 945 | 500.87 | 1.89 (1.77, 2.01) |
| 2017 |  | 14937 | 32530 | 840 | 457.99 | 1.83 (1.71, 1.96) |
| 2018 |  | 14723 | 27772 | 697 | 385.71 | 1.81 (1.68, 1.95) |
| 2019 |  | 15325 | 22487 | 593 | 308.6 | 1.92 (1.77, 2.08) |
| 2020 |  | 13106 | 12267 | 300 | 163.44 | 1.84 (1.63, 2.06) |
| 2000 | Melanoma | 4628 | 74418 | 963 | 746.01 | 1.29 (1.21, 1.38) |
| 2001 |  | 5059 | 79658 | 1039 | 806.24 | 1.29 (1.21, 1.37) |
| 2002 |  | 5064 | 76225 | 1008 | 762.44 | 1.32 (1.24, 1.41) |
| 2003 |  | 4928 | 70734 | 1012 | 706.38 | 1.43 (1.35, 1.52) |
| 2004 |  | 5422 | 75296 | 995 | 758.81 | 1.31 (1.23, 1.40) |
| 2005 |  | 5797 | 76458 | 986 | 757.19 | 1.30 (1.22, 1.39) |
| 2006 |  | 5662 | 70047 | 1092 | 692.58 | 1.58 (1.48, 1.67) |
| 2007 |  | 5736 | 67792 | 1056 | 675.38 | 1.56 (1.47, 1.66) |
| 2008 |  | 5821 | 64777 | 994 | 665.05 | 1.49 (1.40, 1.59) |
| 2009 |  | 5869 | 60793 | 956 | 617.7 | 1.55 (1.45, 1.65) |
| 2010 |  | 6148 | 58673 | 952 | 606.7 | 1.57 (1.47, 1.67) |
| 2011 |  | 5889 | 52285 | 836 | 531.14 | 1.57 (1.47, 1.68) |
| 2012 |  | 6183 | 50074 | 836 | 517.2 | 1.62 (1.51, 1.73) |
| 2013 |  | 6277 | 45226 | 758 | 472.03 | 1.61 (1.49, 1.72) |
| 2014 |  | 6875 | 44444 | 812 | 458.48 | 1.77 (1.65, 1.90) |
| 2015 |  | 7075 | 40035 | 738 | 415.53 | 1.78 (1.65, 1.91) |
| 2016 |  | 6935 | 33150 | 600 | 339.76 | 1.77 (1.63, 1.91) |
| 2017 |  | 7056 | 27873 | 560 | 290.79 | 1.93 (1.77, 2.09) |
| 2018 |  | 6911 | 21097 | 388 | 218.93 | 1.77 (1.60, 1.96) |
| 2019 |  | 7112 | 15407 | 334 | 157.58 | 2.12 (1.90, 2.36) |
| 2020 |  | 5996 | 7375 | 169 | 74.78 | 2.26 (1.93, 2.63) |
| 2000 | Breast | 42804 | 593335 | 9453 | 7685.83 | 1.23 (1.21, 1.25) |
| 2001 |  | 43611 | 592745 | 9168 | 7623.64 | 1.20 (1.18, 1.23) |
| 2002 |  | 43252 | 571420 | 8960 | 7301.2 | 1.23 (1.20, 1.25) |
| 2003 |  | 41275 | 528501 | 8024 | 6561.3 | 1.22 (1.20, 1.25) |
| 2004 |  | 41623 | 514965 | 7649 | 6298.26 | 1.21 (1.19, 1.24) |
| 2005 |  | 41950 | 500763 | 7444 | 6078.3 | 1.22 (1.20, 1.25) |
| 2006 |  | 42386 | 485073 | 7102 | 5860.58 | 1.21 (1.18, 1.24) |
| 2007 |  | 43858 | 477402 | 6674 | 5735.66 | 1.16 (1.14, 1.19) |
| 2008 |  | 44790 | 461365 | 6364 | 5507.67 | 1.16 (1.13, 1.18) |
| 2009 |  | 45647 | 443209 | 6033 | 5266.23 | 1.15 (1.12, 1.17) |
| 2010 |  | 44878 | 406232 | 5300 | 4786.74 | 1.11 (1.08, 1.14) |
| 2011 |  | 46659 | 392934 | 5007 | 4594.3 | 1.09 (1.06, 1.12) |
| 2012 |  | 47134 | 364235 | 4529 | 4228.9 | 1.07 (1.04, 1.10) |
| 2013 |  | 48143 | 337012 | 4145 | 3882.45 | 1.07 (1.04, 1.10) |
| 2014 |  | 48841 | 305974 | 3614 | 3486.44 | 1.04 (1.00, 1.07) |
| 2015 |  | 50623 | 278780 | 3273 | 3152.74 | 1.04 (1.00, 1.07) |
| 2016 |  | 51108 | 239737 | 2754 | 2694.57 | 1.02 (0.98, 1.06) |
| 2017 |  | 51939 | 201050 | 2424 | 2218.41 | 1.09 (1.05, 1.14) |
| 2018 |  | 52551 | 159187 | 1894 | 1735.92 | 1.09 (1.04, 1.14) |
| 2019 |  | 54075 | 116648 | 1501 | 1237.31 | 1.21 (1.15, 1.28) |
| 2020 |  | 48561 | 60342 | 897 | 616.72 | 1.45 (1.36, 1.55) |
| 2000 | Uterine | 7372 | 98652 | 1271 | 1352.13 | 0.94 (0.89, 0.99) |
| 2001 |  | 7803 | 102532 | 1287 | 1396.74 | 0.92 (0.87, 0.97) |
| 2002 |  | 7581 | 95723 | 1210 | 1280.25 | 0.95 (0.89, 1.00) |
| 2003 |  | 7571 | 91725 | 1175 | 1206.02 | 0.97 (0.92, 1.03) |
| 2004 |  | 7747 | 91154 | 1184 | 1171.73 | 1.01 (0.95, 1.07) |
| 2005 |  | 8037 | 91518 | 1150 | 1166.14 | 0.99 (0.93, 1.04) |
| 2006 |  | 8345 | 90874 | 1197 | 1154.6 | 1.04 (0.98, 1.10) |
| 2007 |  | 8725 | 90245 | 1175 | 1135.4 | 1.03 (0.98, 1.10) |
| 2008 |  | 8991 | 87584 | 1148 | 1085.02 | 1.06 (1.00, 1.12) |
| 2009 |  | 9621 | 87553 | 1194 | 1080.05 | 1.11 (1.04, 1.17) |
| 2010 |  | 9905 | 84626 | 1040 | 1041.53 | 1.00 (0.94, 1.06) |
| 2011 |  | 10016 | 78475 | 1003 | 951.33 | 1.05 (0.99, 1.12) |
| 2012 |  | 10766 | 77350 | 1013 | 933.06 | 1.09 (1.02, 1.15) |
| 2013 |  | 10698 | 69709 | 938 | 833.18 | 1.13 (1.05, 1.20) |
| 2014 |  | 11305 | 65837 | 841 | 776.46 | 1.08 (1.01, 1.16) |
| 2015 |  | 11687 | 59754 | 780 | 694.46 | 1.12 (1.05, 1.20) |
| 2016 |  | 12186 | 53121 | 731 | 621.66 | 1.18 (1.09, 1.26) |
| 2017 |  | 12343 | 44422 | 600 | 509.36 | 1.18 (1.09, 1.28) |
| 2018 |  | 12606 | 35788 | 529 | 406.22 | 1.30 (1.19, 1.42) |
| 2019 |  | 12833 | 26122 | 440 | 287.14 | 1.53 (1.39, 1.68) |
| 2020 |  | 11926 | 14066 | 225 | 153.06 | 1.47 (1.28, 1.68) |
|  | **Males** | | | | | |
| 2000 | CRC | 14141 | 127540 | 3059 | 2866.56 | 1.07 (1.03, 1.11) |
| 2001 |  | 14348 | 127000 | 2982 | 2844.02 | 1.05 (1.01, 1.09) |
| 2002 |  | 14175 | 124309 | 2768 | 2694.55 | 1.03 (0.99, 1.07) |
| 2003 |  | 14018 | 121317 | 2675 | 2594.3 | 1.03 (0.99, 1.07) |
| 2004 |  | 14059 | 119649 | 2701 | 2490.15 | 1.08 (1.04, 1.13) |
| 2005 |  | 14020 | 116808 | 2586 | 2361.25 | 1.10 (1.05, 1.14) |
| 2006 |  | 13867 | 112417 | 2520 | 2224.06 | 1.13 (1.09, 1.18) |
| 2007 |  | 14134 | 111834 | 2385 | 2162.72 | 1.10 (1.06, 1.15) |
| 2008 |  | 14236 | 106533 | 2222 | 2006.84 | 1.11 (1.06, 1.15) |
| 2009 |  | 13998 | 100264 | 2097 | 1825.36 | 1.15 (1.10, 1.20) |
| 2010 |  | 13670 | 90732 | 1885 | 1611.99 | 1.17 (1.12, 1.22) |
| 2011 |  | 13571 | 84591 | 1720 | 1471.21 | 1.17 (1.11, 1.23) |
| 2012 |  | 13769 | 80000 | 1646 | 1360.91 | 1.21 (1.15, 1.27) |
| 2013 |  | 13699 | 73471 | 1570 | 1221.37 | 1.29 (1.22, 1.35) |
| 2014 |  | 14351 | 70624 | 1446 | 1134.13 | 1.27 (1.21, 1.34) |
| 2015 |  | 14502 | 63852 | 1298 | 1000.62 | 1.30 (1.23, 1.37) |
| 2016 |  | 14495 | 55583 | 1167 | 852.93 | 1.37 (1.29, 1.45) |
| 2017 |  | 14355 | 46656 | 965 | 700.42 | 1.38 (1.29, 1.47) |
| 2018 |  | 14405 | 37059 | 748 | 546.12 | 1.37 (1.27, 1.47) |
| 2019 |  | 15079 | 28589 | 587 | 406.98 | 1.44 (1.33, 1.56) |
| 2020 |  | 12925 | 14496 | 360 | 200.73 | 1.79 (1.61, 1.99) |
| 2000 | Lung & Bronchus | 16691 | 37973 | 1198 | 857.38 | 1.40 (1.32, 1.48) |
| 2001 |  | 16571 | 38088 | 1260 | 847.7 | 1.49 (1.41, 1.57) |
| 2002 |  | 16177 | 37008 | 1196 | 829.08 | 1.44 (1.36, 1.53) |
| 2003 |  | 16148 | 37680 | 1286 | 830.15 | 1.55 (1.47, 1.64) |
| 2004 |  | 15904 | 36798 | 1173 | 804.7 | 1.46 (1.38, 1.54) |
| 2005 |  | 15708 | 36781 | 1277 | 800.08 | 1.60 (1.51, 1.69) |
| 2006 |  | 15893 | 37152 | 1334 | 796.03 | 1.68 (1.59, 1.77) |
| 2007 |  | 15777 | 37064 | 1254 | 790.6 | 1.59 (1.50, 1.68) |
| 2008 |  | 15707 | 37000 | 1334 | 775.76 | 1.72 (1.63, 1.81) |
| 2009 |  | 15632 | 35773 | 1198 | 745.39 | 1.61 (1.52, 1.70) |
| 2010 |  | 15442 | 34769 | 1144 | 702.41 | 1.63 (1.54, 1.73) |
| 2011 |  | 14910 | 33961 | 1142 | 681.27 | 1.68 (1.58, 1.78) |
| 2012 |  | 15043 | 31988 | 1089 | 648.67 | 1.68 (1.58, 1.78) |
| 2013 |  | 14945 | 31166 | 1127 | 626.61 | 1.80 (1.70, 1.91) |
| 2014 |  | 15138 | 31809 | 1049 | 629.64 | 1.67 (1.57, 1.77) |
| 2015 |  | 15192 | 31421 | 1083 | 616.56 | 1.76 (1.65, 1.86) |
| 2016 |  | 15170 | 30114 | 985 | 594.96 | 1.66 (1.55, 1.76) |
| 2017 |  | 15075 | 27516 | 908 | 539.07 | 1.68 (1.58, 1.80) |
| 2018 |  | 14728 | 23410 | 712 | 448.29 | 1.59 (1.47, 1.71) |
| 2019 |  | 15131 | 19359 | 559 | 366.49 | 1.53 (1.40, 1.66) |
| 2020 |  | 13199 | 11261 | 372 | 208.2 | 1.79 (1.61, 1.98) |
| 2000 | Melanoma | 5730 | 81241 | 1883 | 1366.43 | 1.38 (1.32, 1.44) |
| 2001 |  | 6196 | 85207 | 1971 | 1431.81 | 1.38 (1.32, 1.44) |
| 2002 |  | 6188 | 80680 | 1829 | 1361.64 | 1.34 (1.28, 1.41) |
| 2003 |  | 6104 | 77029 | 1824 | 1304.02 | 1.40 (1.34, 1.46) |
| 2004 |  | 6760 | 81728 | 1981 | 1368.42 | 1.45 (1.38, 1.51) |
| 2005 |  | 7230 | 86045 | 2174 | 1440 | 1.51 (1.45, 1.57) |
| 2006 |  | 7052 | 78899 | 1967 | 1324.19 | 1.49 (1.42, 1.55) |
| 2007 |  | 7237 | 76511 | 2013 | 1292.32 | 1.56 (1.49, 1.63) |
| 2008 |  | 7574 | 75565 | 2012 | 1268.63 | 1.59 (1.52, 1.66) |
| 2009 |  | 7526 | 71362 | 1891 | 1205.18 | 1.57 (1.50, 1.64) |
| 2010 |  | 7601 | 66835 | 1868 | 1127.44 | 1.66 (1.58, 1.73) |
| 2011 |  | 7724 | 62127 | 1715 | 1045.47 | 1.64 (1.56, 1.72) |
| 2012 |  | 8107 | 60788 | 1647 | 1033.06 | 1.59 (1.52, 1.67) |
| 2013 |  | 8419 | 57506 | 1671 | 981.11 | 1.70 (1.62, 1.79) |
| 2014 |  | 8926 | 54759 | 1529 | 921.31 | 1.66 (1.58, 1.74) |
| 2015 |  | 9100 | 49385 | 1400 | 828.67 | 1.69 (1.60, 1.78) |
| 2016 |  | 8996 | 41569 | 1281 | 690.71 | 1.85 (1.75, 1.96) |
| 2017 |  | 9064 | 34846 | 1090 | 592.98 | 1.84 (1.73, 1.95) |
| 2018 |  | 8896 | 26777 | 868 | 444.06 | 1.95 (1.83, 2.09) |
| 2019 |  | 9086 | 19456 | 666 | 322.35 | 2.07 (1.91, 2.23) |
| 2020 |  | 7617 | 9204 | 358 | 151.42 | 2.36 (2.13, 2.62) |
| 2000 | Prostate | 46098 | 599182 | 10537 | 15589.64 | 0.68 (0.66, 0.69) |
| 2001 |  | 47988 | 624330 | 10899 | 15991.77 | 0.68 (0.67, 0.69) |
| 2002 |  | 49313 | 635676 | 10910 | 15959.17 | 0.68 (0.67, 0.70) |
| 2003 |  | 45669 | 570053 | 9491 | 14004.63 | 0.68 (0.66, 0.69) |
| 2004 |  | 46516 | 567466 | 9427 | 13688.4 | 0.69 (0.67, 0.70) |
| 2005 |  | 44915 | 529251 | 8516 | 12550.96 | 0.68 (0.66, 0.69) |
| 2006 |  | 50012 | 577558 | 9035 | 13440.66 | 0.67 (0.66, 0.69) |
| 2007 |  | 53050 | 591196 | 9171 | 13462.75 | 0.68 (0.67, 0.70) |
| 2008 |  | 50482 | 533610 | 8083 | 11809.55 | 0.68 (0.67, 0.70) |
| 2009 |  | 51046 | 513452 | 7585 | 11070.86 | 0.69 (0.67, 0.70) |
| 2010 |  | 49810 | 467626 | 6830 | 9945.98 | 0.69 (0.67, 0.70) |
| 2011 |  | 50476 | 441132 | 6310 | 9251.81 | 0.68 (0.67, 0.70) |
| 2012 |  | 43397 | 343706 | 4691 | 7062.15 | 0.66 (0.65, 0.68) |
| 2013 |  | 42380 | 303680 | 4163 | 6166.12 | 0.68 (0.65, 0.70) |
| 2014 |  | 39828 | 252848 | 3456 | 5036.39 | 0.69 (0.66, 0.71) |
| 2015 |  | 42818 | 238365 | 3302 | 4696.29 | 0.70 (0.68, 0.73) |
| 2016 |  | 45440 | 216510 | 2953 | 4243.97 | 0.70 (0.67, 0.72) |
| 2017 |  | 48750 | 190951 | 2615 | 3684.36 | 0.71 (0.68, 0.74) |
| 2018 |  | 49662 | 151862 | 2104 | 2885.84 | 0.73 (0.70, 0.76) |
| 2019 |  | 52722 | 114696 | 1614 | 2129.96 | 0.76 (0.72, 0.80) |
| 2020 |  | 46854 | 58768 | 957 | 1077.66 | 0.89 (0.83, 0.95) |
| 2000 | Urinary bladder | 8139 | 80431 | 2703 | 1940.82 | 1.39 (1.34, 1.45) |
| 2001 |  | 8196 | 80209 | 2587 | 1929.7 | 1.34 (1.29, 1.39) |
| 2002 |  | 8120 | 78859 | 2646 | 1883.01 | 1.41 (1.35, 1.46) |
| 2003 |  | 8362 | 79357 | 2704 | 1874.85 | 1.44 (1.39, 1.50) |
| 2004 |  | 8718 | 80007 | 2748 | 1874.88 | 1.47 (1.41, 1.52) |
| 2005 |  | 8546 | 75949 | 2757 | 1758.03 | 1.57 (1.51, 1.63) |
| 2006 |  | 8650 | 74610 | 2549 | 1733.76 | 1.47 (1.41, 1.53) |
| 2007 |  | 8794 | 73703 | 2603 | 1681.79 | 1.55 (1.49, 1.61) |
| 2008 |  | 8775 | 69511 | 2455 | 1559.57 | 1.57 (1.51, 1.64) |
| 2009 |  | 8665 | 65561 | 2390 | 1454.55 | 1.64 (1.58, 1.71) |
| 2010 |  | 9145 | 65411 | 2393 | 1448.3 | 1.65 (1.59, 1.72) |
| 2011 |  | 8841 | 59467 | 2215 | 1299.05 | 1.71 (1.63, 1.78) |
| 2012 |  | 9123 | 56696 | 2151 | 1245.11 | 1.73 (1.66, 1.80) |
| 2013 |  | 9104 | 52611 | 2042 | 1147.41 | 1.78 (1.70, 1.86) |
| 2014 |  | 9297 | 48781 | 1975 | 1045.69 | 1.89 (1.81, 1.97) |
| 2015 |  | 9452 | 44314 | 1697 | 941.14 | 1.80 (1.72, 1.89) |
| 2016 |  | 9538 | 38797 | 1648 | 812.52 | 2.03 (1.93, 2.13) |
| 2017 |  | 9689 | 33167 | 1493 | 695.57 | 2.15 (2.04, 2.26) |
| 2018 |  | 9353 | 25635 | 1194 | 533.23 | 2.24 (2.11, 2.37) |
| 2019 |  | 9565 | 18788 | 1054 | 386.48 | 2.73 (2.56, 2.90) |
| 2020 |  | 9165 | 10792 | 745 | 215.22 | 3.46 (3.22, 3.72) |

| **Table S4. Risk of Subsequent Primary Cancer After Index Primary Cancers by Stage of the Index Cancer in the 17 SEER Registries From 2000 to 2021** | | | | | | | | | | | | |
| --- | --- | --- | --- | --- | --- | --- | --- | --- | --- | --- | --- | --- |
| Year of Index Primary Cancer | **Females** | | | | | |  | **Males** | | | | |
|  | Stage of Index Primary Cancer | # of Index Primary Cancer | Person-Years of Follow Up | # of Subsequent Primary Cancer | | Observed/  Expected Ratio  (95% CI) |  | # of Index Primary Cancer | Person-Years of Follow Up | # of Subsequent Primary Cancer | | Observed/  Expected Ratio  (95% CI) |
|  |  |  |  | Observed | Expected |  |  |  |  | Observed | Expected |  |
| 2004 | Localized | 59850 | 755037 | 11264 | 9082.4 | 1.24 (1.22, 1.26) |  | 70682 | 816608 | 15666 | 17392.6 | 0.90 (0.89, 0.91) |
| 2005 |  | 61417 | 746300 | 10937 | 8878.4 | 1.23 (1.21, 1.26) |  | 70210 | 786740 | 15303 | 16421.6 | 0.93 (0.92, 0.95) |
| 2006 |  | 62094 | 720520 | 11034 | 8558.5 | 1.29 (1.27, 1.31) |  | 75231 | 822285 | 15594 | 17118.2 | 0.91 (0.90, 0.93) |
| 2007 |  | 63998 | 708393 | 10380 | 8368.2 | 1.24 (1.22, 1.26) |  | 78483 | 824824 | 15502 | 16894.0 | 0.92 (0.90, 0.93) |
| 2008 |  | 66050 | 690425 | 10173 | 8107.4 | 1.25 (1.23, 1.28) |  | 77061 | 766519 | 14257 | 15167.9 | 0.94 (0.92, 0.96) |
| 2009 |  | 67922 | 669341 | 9672 | 7779.3 | 1.24 (1.22, 1.27) |  | 77162 | 728854 | 13574 | 14125.7 | 0.96 (0.94, 0.98) |
| 2010 |  | 67616 | 621835 | 8903 | 7201.3 | 1.24 (1.21, 1.26) |  | 76062 | 672250 | 12377 | 12812.2 | 0.97 (0.95, 0.98) |
| 2011 |  | 68976 | 586158 | 8506 | 6750.1 | 1.26 (1.23, 1.29) |  | 76958 | 631660 | 11592 | 11903.3 | 0.97 (0.96, 0.99) |
| 2012 |  | 71300 | 554148 | 7847 | 6343.2 | 1.24 (1.21, 1.26) |  | 71479 | 534136 | 9778 | 9766.3 | 1.00 (0.98, 1.02) |
| 2013 |  | 72042 | 506951 | 7342 | 5782.8 | 1.27 (1.24, 1.30) |  | 70969 | 481467 | 9310 | 8716.0 | 1.07 (1.05, 1.09) |
| 2014 |  | 74523 | 468481 | 6785 | 5280.4 | 1.28 (1.25, 1.32) |  | 70138 | 423654 | 8171 | 7479.9 | 1.09 (1.07, 1.12) |
| 2015 |  | 76824 | 423369 | 6072 | 4720.6 | 1.29 (1.25, 1.32) |  | 72587 | 386806 | 7402 | 6767.9 | 1.09 (1.07, 1.12) |
| 2016 |  | 77701 | 364372 | 5178 | 4060.5 | 1.28 (1.24, 1.31) |  | 72158 | 329530 | 6703 | 5739.4 | 1.17 (1.14, 1.20) |
| 2017 |  | 78076 | 301006 | 4407 | 3317.3 | 1.33 (1.29, 1.37) |  | 74261 | 281474 | 5731 | 4875.0 | 1.18 (1.15, 1.21) |
| 2018 |  | 79260 | 238203 | 3521 | 2609.9 | 1.35 (1.30, 1.39) |  | 77464 | 229479 | 4699 | 3926.4 | 1.20 (1.16, 1.23) |
| 2019 |  | 81201 | 173194 | 2726 | 1857.4 | 1.47 (1.41, 1.52) |  | 80204 | 169577 | 3603 | 2856.9 | 1.26 (1.22, 1.30) |
| 2020 |  | 71330 | 87489 | 1489 | 918.2 | 1.62 (1.54, 1.71) |  | 70117 | 85853 | 2149 | 1422.8 | 1.51 (1.45, 1.58) |
| 2004 | Regional | 33671 | 315820 | 4657 | 3590.9 | 1.30 (1.26, 1.33) |  | 26631 | 219963 | 4331 | 4060.3 | 1.07 (1.04, 1.10) |
| 2005 |  | 33884 | 310699 | 4626 | 3527.6 | 1.31 (1.27, 1.35) |  | 26560 | 211892 | 4222 | 3842.7 | 1.10 (1.07, 1.13) |
| 2006 |  | 34361 | 305353 | 4583 | 3446.3 | 1.33 (1.29, 1.37) |  | 26776 | 208168 | 4227 | 3704.9 | 1.14 (1.11, 1.18) |
| 2007 |  | 34946 | 295608 | 4393 | 3313.3 | 1.33 (1.29, 1.37) |  | 28209 | 214789 | 4256 | 3781.4 | 1.13 (1.09, 1.16) |
| 2008 |  | 35617 | 287910 | 4203 | 3187.0 | 1.32 (1.28, 1.36) |  | 28105 | 203831 | 4138 | 3521.3 | 1.18 (1.14, 1.21) |
| 2009 |  | 35973 | 277652 | 4210 | 3062.9 | 1.37 (1.33, 1.42) |  | 28745 | 199890 | 3905 | 3384.3 | 1.15 (1.12, 1.19) |
| 2010 |  | 35411 | 256971 | 3729 | 2806.2 | 1.33 (1.29, 1.37) |  | 28336 | 185601 | 3833 | 3110.2 | 1.23 (1.19, 1.27) |
| 2011 |  | 36185 | 246645 | 3526 | 2673.6 | 1.32 (1.28, 1.36) |  | 28910 | 176557 | 3549 | 2931.4 | 1.21 (1.17, 1.25) |
| 2012 |  | 36445 | 230222 | 3271 | 2478.2 | 1.32 (1.28, 1.37) |  | 28722 | 161616 | 3231 | 2642.3 | 1.22 (1.18, 1.27) |
| 2013 |  | 36308 | 209369 | 3068 | 2233.3 | 1.37 (1.33, 1.42) |  | 28732 | 148895 | 3086 | 2402.1 | 1.28 (1.24, 1.33) |
| 2014 |  | 37365 | 194730 | 2731 | 2062.5 | 1.32 (1.27, 1.37) |  | 29546 | 139621 | 2944 | 2192.9 | 1.34 (1.29, 1.39) |
| 2015 |  | 38152 | 177548 | 2595 | 1864.7 | 1.39 (1.34, 1.45) |  | 30892 | 131588 | 2832 | 2072.5 | 1.37 (1.32, 1.42) |
| 2016 |  | 38523 | 153850 | 2281 | 1629.2 | 1.40 (1.34, 1.46) |  | 32130 | 120198 | 2546 | 1904.7 | 1.34 (1.29, 1.39) |
| 2017 |  | 38359 | 129413 | 1914 | 1354.3 | 1.41 (1.35, 1.48) |  | 32482 | 102539 | 2143 | 1602.1 | 1.34 (1.28, 1.40) |
| 2018 |  | 37792 | 102281 | 1601 | 1051.2 | 1.52 (1.45, 1.60) |  | 31924 | 81886 | 1832 | 1258.7 | 1.46 (1.39, 1.52) |
| 2019 |  | 38797 | 76657 | 1250 | 777.4 | 1.61 (1.52, 1.70) |  | 33552 | 63700 | 1486 | 971.3 | 1.53 (1.45, 1.61) |
| 2020 |  | 35633 | 41632 | 771 | 411.5 | 1.87 (1.74, 2.01) |  | 30702 | 34996 | 1000 | 523.9 | 1.91 (1.79, 2.03) |
| 2004 | Distant | 28138 | 124261 | 1919 | 1475.9 | 1.30 (1.24, 1.36) |  | 31410 | 132010 | 2640 | 2189.4 | 1.21 (1.16, 1.25) |
| 2005 |  | 28664 | 124653 | 1907 | 1475.1 | 1.29 (1.24, 1.35) |  | 31938 | 132731 | 2726 | 2151.8 | 1.27 (1.22, 1.32) |
| 2006 |  | 29117 | 123949 | 1929 | 1481.3 | 1.30 (1.24, 1.36) |  | 32543 | 131862 | 2773 | 2133.8 | 1.30 (1.25, 1.35) |
| 2007 |  | 29871 | 126564 | 1977 | 1495.7 | 1.32 (1.26, 1.38) |  | 33016 | 134178 | 2826 | 2170.3 | 1.30 (1.25, 1.35) |
| 2008 |  | 30536 | 126656 | 2072 | 1490.8 | 1.39 (1.33, 1.45) |  | 33851 | 132982 | 2853 | 2134.7 | 1.34 (1.29, 1.39) |
| 2009 |  | 31161 | 122586 | 2097 | 1444.2 | 1.45 (1.39, 1.52) |  | 34483 | 131852 | 2896 | 2115.7 | 1.37 (1.32, 1.42) |
| 2010 |  | 31523 | 121927 | 2005 | 1447.0 | 1.39 (1.33, 1.45) |  | 35524 | 129700 | 2949 | 2076.6 | 1.42 (1.37, 1.47) |
| 2011 |  | 31609 | 116304 | 2020 | 1362.2 | 1.48 (1.42, 1.55) |  | 35203 | 124312 | 2966 | 1991.5 | 1.49 (1.44, 1.54) |
| 2012 |  | 32251 | 112972 | 2026 | 1329.0 | 1.52 (1.46, 1.59) |  | 36851 | 123401 | 2891 | 1981.8 | 1.46 (1.41, 1.51) |
| 2013 |  | 32892 | 108883 | 1850 | 1283.9 | 1.44 (1.38, 1.51) |  | 37337 | 118392 | 2833 | 1888.3 | 1.50 (1.45, 1.56) |
| 2014 |  | 33542 | 104792 | 1788 | 1230.3 | 1.45 (1.39, 1.52) |  | 38474 | 115081 | 2676 | 1833.6 | 1.46 (1.40, 1.52) |
| 2015 |  | 34791 | 99607 | 1676 | 1169.0 | 1.43 (1.37, 1.50) |  | 39002 | 107332 | 2495 | 1729.0 | 1.44 (1.39, 1.50) |
| 2016 |  | 34165 | 88467 | 1530 | 1040.0 | 1.47 (1.40, 1.55) |  | 39087 | 98095 | 2330 | 1568.7 | 1.49 (1.43, 1.55) |
| 2017 |  | 34370 | 77663 | 1334 | 911.7 | 1.46 (1.39, 1.54) |  | 39595 | 87491 | 2063 | 1405.1 | 1.47 (1.41, 1.53) |
| 2018 |  | 35013 | 66889 | 1210 | 779.0 | 1.55 (1.47, 1.64) |  | 39586 | 73247 | 1723 | 1173.6 | 1.47 (1.40, 1.54) |
| 2019 |  | 35989 | 52714 | 999 | 602.6 | 1.66 (1.56, 1.76) |  | 41013 | 58875 | 1384 | 934.0 | 1.48 (1.40, 1.56) |
| 2020 |  | 33897 | 31674 | 643 | 354.5 | 1.81 (1.68, 1.96) |  | 38392 | 35106 | 978 | 549.3 | 1.78 (1.67, 1.90) |

| **Table S5. Risk of Subsequent Primary Cancer After Index Primary Cancers by Age at Diagnosis of the Index Cancer in the 17 SEER Registries From 2000 to 2021** | | | | | | | | | | | | | | |  |  |
| --- | --- | --- | --- | --- | --- | --- | --- | --- | --- | --- | --- | --- | --- | --- | --- | --- |
| Year of Index Primary Cancer | **Females** | | | | | |  | | **Males** | | | | | | |  |
|  | Age at Diagnosis | # of Index Primary Cancer | Person-Years of Follow Up | # of Subsequent Primary Cancer | | Observed/  Expected Ratio  (95% CI) | |  | | # of Index Primary Cancer | Person-Years of Follow Up | # of Subsequent Primary Cancer | | Observed/  Expected Ratio  (95% CI) | | |
|  |  |  |  | Observed | Expected |  |  |  | |  |  | Observed | Expected |  |  |  |
| 2000 | 0-14 | 1111 | 18322 | 35 | 6.2 | 5.62 (3.91, 7.81) | |  | | 1280 | 21563 | 44 | 6.3 | 6.98 (5.07, 9.38) | | |
| 2001 |  | 1210 | 19499 | 43 | 6.3 | 6.82 (4.93, 9.18) | |  | | 1358 | 21521 | 41 | 6.1 | 6.75 (4.85, 9.16) | | |
| 2002 |  | 1113 | 17118 | 27 | 5.1 | 5.33 (3.51, 7.76) | |  | | 1396 | 20710 | 47 | 5.5 | 8.57 (6.30, 11.40) | | |
| 2003 |  | 1092 | 16588 | 37 | 4.6 | 8.04 (5.66, 11.09) | |  | | 1251 | 17933 | 31 | 4.6 | 6.69 (4.55, 9.50) | | |
| 2004 |  | 1164 | 16460 | 38 | 4.3 | 8.77 (6.21, 12.04) | |  | | 1397 | 19661 | 30 | 4.9 | 6.12 (4.13, 8.74) | | |
| 2005 |  | 1167 | 15367 | 39 | 4.0 | 9.81 (6.98, 13.41) | |  | | 1427 | 18644 | 44 | 4.5 | 9.80 (7.12, 13.15) | | |
| 2006 |  | 1110 | 13861 | 36 | 3.2 | 11.16 (7.82, 15.46) | |  | | 1345 | 16851 | 44 | 4.0 | 10.97 (7.97, 14.72) | | |
| 2007 |  | 1224 | 14179 | 33 | 3.1 | 10.52 (7.24, 14.78) | |  | | 1437 | 16338 | 34 | 3.7 | 9.12 (6.31, 12.74) | | |
| 2008 |  | 1163 | 12323 | 31 | 2.7 | 11.63 (7.90, 16.51) | |  | | 1463 | 15676 | 38 | 3.5 | 10.99 (7.78, 15.09) | | |
| 2009 |  | 1259 | 12477 | 24 | 2.6 | 9.27 (5.94, 13.79) | |  | | 1449 | 14126 | 25 | 3.1 | 8.08 (5.23, 11.93) | | |
| 2010 |  | 1256 | 11575 | 24 | 2.3 | 10.35 (6.63, 15.39) | |  | | 1529 | 13798 | 20 | 2.9 | 7.02 (4.29, 10.85) | | |
| 2011 |  | 1314 | 10840 | 23 | 2.1 | 11.05 (7.01, 16.58) | |  | | 1431 | 11753 | 37 | 2.4 | 15.67 (11.04, 21.60) | | |
| 2012 |  | 1248 | 9265 | 16 | 1.7 | 9.27 (5.30, 15.05) | |  | | 1454 | 10737 | 19 | 2.2 | 8.85 (5.33, 13.82) | | |
| 2013 |  | 1291 | 8419 | 22 | 1.5 | 14.37 (9.01, 21.76) | |  | | 1451 | 9741 | 27 | 1.9 | 14.04 (9.25, 20.43) | | |
| 2014 |  | 1289 | 7624 | 26 | 1.4 | 19.04 (12.44, 27.90) | |  | | 1502 | 8932 | 27 | 1.7 | 15.62 (10.30, 22.73) | | |
| 2015 |  | 1408 | 7241 | 17 | 1.3 | 13.34 (7.77, 21.36) | |  | | 1537 | 7836 | 20 | 1.5 | 13.28 (8.11, 20.50) | | |
| 2016 |  | 1300 | 5703 | 18 | 1.0 | 18.22 (10.80, 28.80) | |  | | 1481 | 6469 | 28 | 1.2 | 23.06 (15.32, 33.32) | | |
| 2017 |  | 1248 | 4532 | 5 | 0.8 | 6.32 (2.05, 14.74) | |  | | 1482 | 5346 | 15 | 1.0 | 14.91 (8.35, 24.59) | | |
| 2018 |  | 1237 | 3430 | 9 | 0.6 | 15.25 (6.97, 28.95) | |  | | 1470 | 4058 | 14 | 0.8 | 18.36 (10.04, 30.81) | | |
| 2019 |  | 1132 | 2185 | 10 | 0.4 | 27.00 (12.95, 49.65) | |  | | 1398 | 2777 | 6 | 0.5 | 11.61 (4.26, 25.26) | | |
| 2020 |  | 1172 | 1326 | 5 | 0.2 | 22.53 (7.32, 52.59) | |  | | 1345 | 1545 | 4 | 0.3 | 13.83 (3.77, 35.42) | | |
| 2000 | 15-39 | 10036 | 161615 | 1047 | 560.5 | 1.87 (1.76, 1.98) | |  | | 6833 | 101377 | 574 | 220.4 | 2.60 (2.40, 2.83) | | |
| 2001 |  | 10239 | 159839 | 1029 | 532.6 | 1.93 (1.82, 2.05) | |  | | 6858 | 100051 | 446 | 202.7 | 2.20 (2.00, 2.41) | | |
| 2002 |  | 10158 | 152689 | 1063 | 487.3 | 2.18 (2.05, 2.32) | |  | | 6781 | 93695 | 449 | 178.3 | 2.52 (2.29, 2.76) | | |
| 2003 |  | 10173 | 145781 | 966 | 447.3 | 2.16 (2.03, 2.30) | |  | | 6776 | 90583 | 419 | 158.3 | 2.65 (2.40, 2.91) | | |
| 2004 |  | 10358 | 142084 | 840 | 421.9 | 1.99 (1.86, 2.13) | |  | | 6841 | 87168 | 412 | 140.6 | 2.93 (2.65, 3.23) | | |
| 2005 |  | 10532 | 137288 | 804 | 391.0 | 2.06 (1.92, 2.20) | |  | | 6867 | 84178 | 358 | 128.4 | 2.79 (2.51, 3.09) | | |
| 2006 |  | 10489 | 129727 | 826 | 356.0 | 2.32 (2.16, 2.48) | |  | | 6784 | 77696 | 379 | 109.7 | 3.46 (3.12, 3.82) | | |
| 2007 |  | 10604 | 123257 | 712 | 324.9 | 2.19 (2.03, 2.36) | |  | | 7002 | 76611 | 282 | 99.4 | 2.84 (2.52, 3.19) | | |
| 2008 |  | 11094 | 120729 | 664 | 306.4 | 2.17 (2.01, 2.34) | |  | | 7133 | 73131 | 313 | 90.0 | 3.48 (3.10, 3.89) | | |
| 2009 |  | 11109 | 114044 | 621 | 275.0 | 2.26 (2.08, 2.44) | |  | | 7065 | 67817 | 305 | 78.9 | 3.87 (3.44, 4.32) | | |
| 2010 |  | 10812 | 101559 | 540 | 235.2 | 2.30 (2.11, 2.50) | |  | | 7096 | 63212 | 276 | 69.2 | 3.99 (3.53, 4.49) | | |
| 2011 |  | 11173 | 96534 | 546 | 213.4 | 2.56 (2.35, 2.78) | |  | | 7127 | 57912 | 295 | 60.2 | 4.90 (4.35, 5.49) | | |
| 2012 |  | 11519 | 90823 | 498 | 192.5 | 2.59 (2.36, 2.82) | |  | | 7166 | 53786 | 213 | 52.9 | 4.02 (3.50, 4.60) | | |
| 2013 |  | 11252 | 79948 | 408 | 163.3 | 2.50 (2.26, 2.75) | |  | | 7296 | 49077 | 204 | 46.0 | 4.44 (3.85, 5.09) | | |
| 2014 |  | 11866 | 74428 | 448 | 146.3 | 3.06 (2.79, 3.36) | |  | | 7767 | 46576 | 224 | 41.9 | 5.35 (4.67, 6.10) | | |
| 2015 |  | 12373 | 67821 | 347 | 127.6 | 2.72 (2.44, 3.02) | |  | | 7643 | 40005 | 186 | 34.8 | 5.34 (4.60, 6.16) | | |
| 2016 |  | 12544 | 58167 | 306 | 105.3 | 2.91 (2.59, 3.25) | |  | | 8044 | 35607 | 184 | 29.4 | 6.26 (5.39, 7.23) | | |
| 2017 |  | 12259 | 46698 | 266 | 81.4 | 3.27 (2.89, 3.69) | |  | | 7858 | 28603 | 131 | 23.2 | 5.65 (4.72, 6.70) | | |
| 2018 |  | 12510 | 37065 | 232 | 61.2 | 3.79 (3.32, 4.31) | |  | | 7717 | 22032 | 133 | 17.4 | 7.66 (6.42, 9.08) | | |
| 2019 |  | 12676 | 26741 | 164 | 42.3 | 3.88 (3.31, 4.52) | |  | | 7969 | 16201 | 92 | 12.1 | 7.59 (6.12, 9.31) | | |
| 2020 |  | 11717 | 14363 | 99 | 21.6 | 4.59 (3.73, 5.59) | |  | | 7341 | 8701 | 57 | 6.2 | 9.20 (6.97, 11.92) | | |
| 2000 | 40-49 | 17303 | 262797 | 2847 | 1951.6 | 1.46 (1.41, 1.51) | |  | | 10480 | 128928 | 1630 | 1078.0 | 1.51 (1.44, 1.59) | | |
| 2001 |  | 17885 | 262205 | 2756 | 1892.8 | 1.46 (1.40, 1.51) | |  | | 11010 | 134474 | 1551 | 1081.3 | 1.43 (1.36, 1.51) | | |
| 2002 |  | 17944 | 252207 | 2683 | 1773.9 | 1.51 (1.46, 1.57) | |  | | 11002 | 129570 | 1493 | 997.6 | 1.50 (1.42, 1.57) | | |
| 2003 |  | 18361 | 248596 | 2591 | 1699.5 | 1.52 (1.47, 1.58) | |  | | 11062 | 125395 | 1388 | 916.3 | 1.51 (1.44, 1.60) | | |
| 2004 |  | 18841 | 244495 | 2463 | 1633.1 | 1.51 (1.45, 1.57) | |  | | 11460 | 127678 | 1313 | 887.5 | 1.48 (1.40, 1.56) | | |
| 2005 |  | 18970 | 235344 | 2422 | 1533.3 | 1.58 (1.52, 1.64) | |  | | 11619 | 123411 | 1356 | 808.6 | 1.68 (1.59, 1.77) | | |
| 2006 |  | 19109 | 224539 | 2300 | 1424.7 | 1.61 (1.55, 1.68) | |  | | 11692 | 119325 | 1243 | 739.7 | 1.68 (1.59, 1.78) | | |
| 2007 |  | 19114 | 211679 | 1949 | 1308.7 | 1.49 (1.42, 1.56) | |  | | 11699 | 113212 | 1160 | 665.3 | 1.74 (1.64, 1.85) | | |
| 2008 |  | 19046 | 197945 | 1850 | 1191.0 | 1.55 (1.48, 1.63) | |  | | 11736 | 108441 | 1076 | 601.5 | 1.79 (1.68, 1.90) | | |
| 2009 |  | 19433 | 190368 | 1796 | 1115.7 | 1.61 (1.54, 1.69) | |  | | 11544 | 99839 | 965 | 521.2 | 1.85 (1.74, 1.97) | | |
| 2010 |  | 18804 | 170548 | 1558 | 966.3 | 1.61 (1.53, 1.69) | |  | | 11141 | 88539 | 856 | 430.4 | 1.99 (1.86, 2.13) | | |
| 2011 |  | 18995 | 157514 | 1387 | 867.1 | 1.60 (1.52, 1.69) | |  | | 10950 | 81509 | 819 | 370.5 | 2.21 (2.06, 2.37) | | |
| 2012 |  | 18712 | 142417 | 1192 | 763.3 | 1.56 (1.47, 1.65) | |  | | 10766 | 73167 | 637 | 309.5 | 2.06 (1.90, 2.22) | | |
| 2013 |  | 18565 | 128235 | 1082 | 669.2 | 1.62 (1.52, 1.72) | |  | | 10115 | 62096 | 619 | 243.5 | 2.54 (2.35, 2.75) | | |
| 2014 |  | 18581 | 113702 | 979 | 575.1 | 1.70 (1.60, 1.81) | |  | | 9764 | 53984 | 523 | 196.9 | 2.66 (2.43, 2.89) | | |
| 2015 |  | 18710 | 100081 | 862 | 492.5 | 1.75 (1.64, 1.87) | |  | | 9938 | 48474 | 466 | 163.0 | 2.86 (2.61, 3.13) | | |
| 2016 |  | 18120 | 82168 | 722 | 392.1 | 1.84 (1.71, 1.98) | |  | | 9682 | 40306 | 383 | 127.2 | 3.01 (2.72, 3.33) | | |
| 2017 |  | 18633 | 69852 | 590 | 322.8 | 1.83 (1.68, 1.98) | |  | | 9645 | 33117 | 345 | 97.9 | 3.52 (3.16, 3.92) | | |
| 2018 |  | 18449 | 53997 | 499 | 241.7 | 2.06 (1.89, 2.25) | |  | | 9578 | 26228 | 270 | 72.4 | 3.73 (3.30, 4.20) | | |
| 2019 |  | 18890 | 39447 | 370 | 170.8 | 2.17 (1.95, 2.40) | |  | | 9648 | 19013 | 190 | 48.7 | 3.90 (3.36, 4.49) | | |
| 2020 |  | 17209 | 20867 | 250 | 87.6 | 2.85 (2.51, 3.23) | |  | | 8587 | 9961 | 126 | 23.6 | 5.33 (4.44, 6.35) | | |
| 2000 | 50-64 | 36770 | 472680 | 7635 | 6223.5 | 1.23 (1.20, 1.25) | |  | | 42166 | 493692 | 9353 | 10007.3 | 0.93 (0.92, 0.95) | | |
| 2001 |  | 38723 | 488999 | 7784 | 6344.9 | 1.23 (1.20, 1.25) | |  | | 44559 | 514130 | 9590 | 10218.6 | 0.94 (0.92, 0.96) | | |
| 2002 |  | 39891 | 486526 | 7723 | 6202.8 | 1.25 (1.22, 1.27) | |  | | 46421 | 528846 | 9762 | 10279.8 | 0.95 (0.93, 0.97) | | |
| 2003 |  | 39490 | 460803 | 7313 | 5758.8 | 1.27 (1.24, 1.30) | |  | | 46258 | 505172 | 9359 | 9555.4 | 0.98 (0.96, 1.00) | | |
| 2004 |  | 40622 | 457784 | 7070 | 5605.7 | 1.26 (1.23, 1.29) | |  | | 48135 | 507785 | 9101 | 9351.5 | 0.97 (0.95, 0.99) | | |
| 2005 |  | 42322 | 459969 | 6957 | 5541.5 | 1.26 (1.23, 1.29) | |  | | 48933 | 494879 | 9227 | 8885.7 | 1.04 (1.02, 1.06) | | |
| 2006 |  | 43293 | 449571 | 6815 | 5344.2 | 1.28 (1.25, 1.31) | |  | | 52536 | 516485 | 9118 | 9072.2 | 1.01 (0.98, 1.03) | | |
| 2007 |  | 45305 | 448849 | 6592 | 5227.6 | 1.26 (1.23, 1.29) | |  | | 55593 | 527711 | 9306 | 9028.6 | 1.03 (1.01, 1.05) | | |
| 2008 |  | 46916 | 439960 | 6368 | 5008.3 | 1.27 (1.24, 1.30) | |  | | 56079 | 499068 | 8696 | 8216.9 | 1.06 (1.04, 1.08) | | |
| 2009 |  | 48233 | 423970 | 6204 | 4740.1 | 1.31 (1.28, 1.34) | |  | | 57600 | 484204 | 8281 | 7714.2 | 1.07 (1.05, 1.10) | | |
| 2010 |  | 48942 | 401869 | 5622 | 4395.1 | 1.28 (1.25, 1.31) | |  | | 57412 | 447053 | 7726 | 6908.1 | 1.12 (1.09, 1.14) | | |
| 2011 |  | 50044 | 378959 | 5387 | 4073.1 | 1.32 (1.29, 1.36) | |  | | 58369 | 419796 | 7051 | 6309.8 | 1.12 (1.09, 1.14) | | |
| 2012 |  | 51169 | 355232 | 4911 | 3721.1 | 1.32 (1.28, 1.36) | |  | | 55538 | 359564 | 6154 | 5176.7 | 1.19 (1.16, 1.22) | | |
| 2013 |  | 51356 | 322076 | 4418 | 3300.7 | 1.34 (1.30, 1.38) | |  | | 55151 | 323880 | 5627 | 4496.6 | 1.25 (1.22, 1.28) | | |
| 2014 |  | 53438 | 301767 | 4063 | 3023.4 | 1.34 (1.30, 1.39) | |  | | 55898 | 292594 | 5111 | 3887.3 | 1.31 (1.28, 1.35) | | |
| 2015 |  | 54794 | 272773 | 3665 | 2674.9 | 1.37 (1.33, 1.42) | |  | | 56721 | 264905 | 4545 | 3408.7 | 1.33 (1.29, 1.37) | | |
| 2016 |  | 54735 | 233255 | 3106 | 2236.6 | 1.39 (1.34, 1.44) | |  | | 57219 | 231040 | 3994 | 2882.8 | 1.39 (1.34, 1.43) | | |
| 2017 |  | 54448 | 191904 | 2629 | 1796.8 | 1.46 (1.41, 1.52) | |  | | 57888 | 195408 | 3286 | 2354.9 | 1.40 (1.35, 1.44) | | |
| 2018 |  | 53463 | 148792 | 2052 | 1355.0 | 1.51 (1.45, 1.58) | |  | | 57299 | 152987 | 2672 | 1765.7 | 1.51 (1.46, 1.57) | | |
| 2019 |  | 54456 | 108953 | 1593 | 958.7 | 1.66 (1.58, 1.75) | |  | | 57717 | 112302 | 2061 | 1229.6 | 1.68 (1.60, 1.75) | | |
| 2020 |  | 48700 | 57009 | 933 | 484.1 | 1.93 (1.81, 2.05) | |  | | 50912 | 58427 | 1315 | 606.0 | 2.17 (2.05, 2.29) | | |
| 2000 | 65-84 | 51210 | 384116 | 8109 | 7163.0 | 1.13 (1.11, 1.16) | |  | | 67014 | 515450 | 14183 | 15780.8 | 0.90 (0.88, 0.91) | | |
| 2001 |  | 52523 | 392785 | 8175 | 7305.5 | 1.12 (1.09, 1.14) | |  | | 69038 | 536171 | 14759 | 16269.5 | 0.91 (0.89, 0.92) | | |
| 2002 |  | 52007 | 383553 | 8045 | 7118.1 | 1.13 (1.11, 1.16) | |  | | 68388 | 532375 | 14318 | 15983.9 | 0.90 (0.88, 0.91) | | |
| 2003 |  | 50659 | 363969 | 7709 | 6733.7 | 1.14 (1.12, 1.17) | |  | | 65958 | 496561 | 13646 | 14740.9 | 0.93 (0.91, 0.94) | | |
| 2004 |  | 51061 | 363889 | 7941 | 6710.1 | 1.18 (1.16, 1.21) | |  | | 66899 | 501653 | 13859 | 14696.6 | 0.94 (0.93, 0.96) | | |
| 2005 |  | 50510 | 355794 | 7712 | 6535.5 | 1.18 (1.15, 1.21) | |  | | 65371 | 479928 | 13372 | 13913.3 | 0.96 (0.94, 0.98) | | |
| 2006 |  | 51014 | 353469 | 7891 | 6463.8 | 1.22 (1.19, 1.25) | |  | | 67722 | 498635 | 13617 | 14273.6 | 0.95 (0.94, 0.97) | | |
| 2007 |  | 51810 | 350556 | 7805 | 6364.7 | 1.23 (1.20, 1.25) | |  | | 69613 | 505917 | 13625 | 14262.5 | 0.96 (0.94, 0.97) | | |
| 2008 |  | 52762 | 348895 | 7823 | 6295.8 | 1.24 (1.22, 1.27) | |  | | 68284 | 473366 | 12897 | 13112.8 | 0.98 (0.97, 1.00) | | |
| 2009 |  | 53657 | 343175 | 7561 | 6164.6 | 1.23 (1.20, 1.25) | |  | | 68621 | 460728 | 12463 | 12494.6 | 1.00 (0.98, 1.02) | | |
| 2010 |  | 53444 | 328366 | 7160 | 5862.3 | 1.22 (1.19, 1.25) | |  | | 68757 | 437556 | 11935 | 11701.1 | 1.02 (1.00, 1.04) | | |
| 2011 |  | 53769 | 315505 | 6909 | 5596.8 | 1.23 (1.21, 1.26) | |  | | 68937 | 417287 | 11460 | 11033.6 | 1.04 (1.02, 1.06) | | |
| 2012 |  | 55832 | 308774 | 6711 | 5424.3 | 1.24 (1.21, 1.27) | |  | | 67523 | 372410 | 10265 | 9690.4 | 1.06 (1.04, 1.08) | | |
| 2013 |  | 57124 | 293094 | 6387 | 5095.8 | 1.25 (1.22, 1.28) | |  | | 68397 | 350420 | 9986 | 8976.3 | 1.11 (1.09, 1.13) | | |
| 2014 |  | 58413 | 275060 | 5836 | 4741.7 | 1.23 (1.20, 1.26) | |  | | 68565 | 316846 | 9136 | 8002.0 | 1.14 (1.12, 1.17) | | |
| 2015 |  | 60589 | 255901 | 5485 | 4369.0 | 1.26 (1.22, 1.29) | |  | | 72130 | 302051 | 8551 | 7526.0 | 1.14 (1.11, 1.16) | | |
| 2016 |  | 62566 | 231982 | 4932 | 3924.5 | 1.26 (1.22, 1.29) | |  | | 74799 | 275999 | 8018 | 6798.6 | 1.18 (1.15, 1.21) | | |
| 2017 |  | 63968 | 200078 | 4257 | 3334.7 | 1.28 (1.24, 1.32) | |  | | 78445 | 246596 | 7148 | 5976.7 | 1.20 (1.17, 1.22) | | |
| 2018 |  | 65484 | 165915 | 3557 | 2722.5 | 1.31 (1.26, 1.35) | |  | | 79310 | 200875 | 5770 | 4794.9 | 1.20 (1.17, 1.23) | | |
| 2019 |  | 67407 | 124875 | 2782 | 2002.0 | 1.39 (1.34, 1.44) | |  | | 84364 | 156523 | 4524 | 3651.5 | 1.24 (1.20, 1.28) | | |
| 2020 |  | 61022 | 66918 | 1586 | 1054.0 | 1.50 (1.43, 1.58) | |  | | 77132 | 85221 | 2905 | 1952.6 | 1.49 (1.43, 1.54) | | |
| 2000 | 85+ | 8050 | 24155 | 439 | 476.5 | 0.92 (0.84, 1.01) | |  | | 5763 | 16192 | 450 | 544.9 | 0.83 (0.75, 0.91) | | |
| 2001 |  | 8482 | 25599 | 440 | 504.0 | 0.87 (0.79, 0.96) | |  | | 5987 | 17076 | 520 | 571.0 | 0.91 (0.83, 0.99) | | |
| 2002 |  | 8326 | 24680 | 462 | 485.1 | 0.95 (0.87, 1.04) | |  | | 6115 | 17909 | 499 | 593.7 | 0.84 (0.77, 0.92) | | |
| 2003 |  | 8419 | 25632 | 502 | 503.6 | 1.00 (0.91, 1.09) | |  | | 6080 | 17822 | 567 | 584.6 | 0.97 (0.89, 1.05) | | |
| 2004 |  | 8465 | 25133 | 479 | 492.6 | 0.97 (0.89, 1.06) | |  | | 6412 | 18612 | 583 | 604.1 | 0.97 (0.89, 1.05) | | |
| 2005 |  | 8749 | 26982 | 497 | 525.7 | 0.95 (0.86, 1.03) | |  | | 6275 | 18115 | 541 | 583.4 | 0.93 (0.85, 1.01) | | |
| 2006 |  | 9078 | 27081 | 553 | 523.9 | 1.06 (0.97, 1.15) | |  | | 6561 | 19118 | 624 | 610.6 | 1.02 (0.94, 1.11) | | |
| 2007 |  | 9204 | 28434 | 571 | 545.4 | 1.05 (0.96, 1.14) | |  | | 6858 | 20535 | 667 | 651.0 | 1.02 (0.95, 1.11) | | |
| 2008 |  | 9572 | 29269 | 620 | 555.2 | 1.12 (1.03, 1.21) | |  | | 7061 | 20356 | 657 | 639.6 | 1.03 (0.95, 1.11) | | |
| 2009 |  | 9722 | 29902 | 605 | 559.1 | 1.08 (1.00, 1.17) | |  | | 7085 | 19375 | 646 | 598.3 | 1.08 (1.00, 1.17) | | |
| 2010 |  | 9684 | 28666 | 563 | 531.5 | 1.06 (0.97, 1.15) | |  | | 7206 | 19612 | 671 | 602.0 | 1.11 (1.03, 1.20) | | |
| 2011 |  | 9527 | 29012 | 591 | 534.1 | 1.11 (1.02, 1.20) | |  | | 7073 | 19294 | 665 | 588.8 | 1.13 (1.05, 1.22) | | |
| 2012 |  | 9647 | 27720 | 563 | 507.4 | 1.11 (1.02, 1.21) | |  | | 7046 | 18540 | 639 | 560.3 | 1.14 (1.05, 1.23) | | |
| 2013 |  | 9585 | 26938 | 611 | 489.2 | 1.25 (1.15, 1.35) | |  | | 7195 | 18243 | 619 | 551.5 | 1.12 (1.04, 1.21) | | |
| 2014 |  | 9491 | 25709 | 543 | 463.1 | 1.17 (1.08, 1.28) | |  | | 7219 | 17989 | 593 | 540.5 | 1.10 (1.01, 1.19) | | |
| 2015 |  | 9519 | 23930 | 509 | 426.2 | 1.19 (1.09, 1.30) | |  | | 7381 | 17523 | 614 | 521.7 | 1.18 (1.09, 1.27) | | |
| 2016 |  | 9766 | 22642 | 455 | 401.4 | 1.13 (1.03, 1.24) | |  | | 7548 | 16736 | 559 | 495.5 | 1.13 (1.04, 1.23) | | |
| 2017 |  | 9444 | 19866 | 441 | 350.2 | 1.26 (1.14, 1.38) | |  | | 7568 | 15198 | 521 | 448.7 | 1.16 (1.06, 1.27) | | |
| 2018 |  | 9336 | 16400 | 368 | 285.4 | 1.29 (1.16, 1.43) | |  | | 7465 | 12908 | 467 | 376.2 | 1.24 (1.13, 1.36) | | |
| 2019 |  | 9405 | 12775 | 309 | 216.6 | 1.43 (1.27, 1.59) | |  | | 7635 | 10413 | 413 | 298.7 | 1.38 (1.25, 1.52) | | |
| 2020 |  | 8143 | 6994 | 186 | 117.3 | 1.59 (1.37, 1.83) | |  | | 6729 | 5869 | 236 | 167.3 | 1.41 (1.24, 1.60) | | |

| **Table S6. Risk of Subsequent Primary Cancer After Index Primary Cancers by Race/Ethnicity in the 17 SEER Registries From 2000 to 2021** | | | | | | | | | | | | |
| --- | --- | --- | --- | --- | --- | --- | --- | --- | --- | --- | --- | --- |
| Year of Index primary cancer | **Females** | | | | | |  | **Males** | | | | |
|  | Race/  Ethnicity | # of Index primary cancer | Person-years of follow up | # of subsequent primary cancer | | Observed/  Expected Ratio  (95% CI) |  | # of Index primary cancer | Person-years of follow up | # of subsequent primary cancer | | Observed/  Expected Ratio  (95% CI) |
|  |  |  |  | Observed | Expected |  |  |  |  | Observed | Expected |  |
| 2000 | Non-Hispanic White | 102558 | 989436 | 21662 | 22372.1 | 0.97 (0.96, 0.98) |  | 94955 | 1009680 | 16061 | 13641.5 | 1.18 (1.16, 1.20) |
| 2001 |  | 105514 | 1013738 | 21934 | 22824.6 | 0.96 (0.95, 0.97) |  | 97877 | 1024980 | 16081 | 13752.7 | 1.17 (1.15, 1.19) |
| 2002 |  | 105784 | 1005399 | 21438 | 22396.9 | 0.96 (0.94, 0.97) |  | 96926 | 987073 | 15641 | 13176.5 | 1.19 (1.17, 1.21) |
| 2003 |  | 102777 | 947012 | 20456 | 20628.1 | 0.99 (0.98, 1.01) |  | 94865 | 935059 | 14899 | 12294.3 | 1.21 (1.19, 1.23) |
| 2004 |  | 104848 | 943834 | 20239 | 20238.5 | 1.00 (0.99, 1.01) |  | 95671 | 917168 | 14436 | 11959.9 | 1.21 (1.19, 1.23) |
| 2005 |  | 103843 | 908092 | 20031 | 19119.8 | 1.05 (1.03, 1.06) |  | 96263 | 896041 | 14074 | 11609.0 | 1.21 (1.19, 1.23) |
| 2006 |  | 108302 | 930079 | 19900 | 19507.8 | 1.02 (1.01, 1.03) |  | 97130 | 870131 | 14053 | 11259.8 | 1.25 (1.23, 1.27) |
| 2007 |  | 111506 | 930407 | 19965 | 19338.1 | 1.03 (1.02, 1.05) |  | 98107 | 842176 | 13332 | 10852.8 | 1.23 (1.21, 1.25) |
| 2008 |  | 109913 | 868265 | 18651 | 17566.6 | 1.06 (1.05, 1.08) |  | 99538 | 816275 | 13110 | 10466.8 | 1.25 (1.23, 1.27) |
| 2009 |  | 109943 | 826577 | 17755 | 16409.3 | 1.08 (1.07, 1.10) |  | 100660 | 784189 | 12488 | 9991.4 | 1.25 (1.23, 1.27) |
| 2010 |  | 109091 | 766976 | 16906 | 15040.3 | 1.12 (1.11, 1.14) |  | 99365 | 727689 | 11521 | 9256.1 | 1.24 (1.22, 1.27) |
| 2011 |  | 108829 | 718767 | 15894 | 13982.2 | 1.14 (1.12, 1.15) |  | 99352 | 681330 | 10815 | 8622.2 | 1.25 (1.23, 1.28) |
| 2012 |  | 104767 | 627134 | 13958 | 11938.4 | 1.17 (1.15, 1.19) |  | 100538 | 637791 | 10126 | 8045.9 | 1.26 (1.23, 1.28) |
| 2013 |  | 104285 | 571709 | 13159 | 10807.6 | 1.22 (1.20, 1.24) |  | 100366 | 582808 | 9345 | 7326.4 | 1.28 (1.25, 1.30) |
| 2014 |  | 104588 | 516001 | 12091 | 9564.6 | 1.26 (1.24, 1.29) |  | 102234 | 537928 | 8575 | 6715.1 | 1.28 (1.25, 1.30) |
| 2015 |  | 106906 | 473077 | 11045 | 8745.6 | 1.26 (1.24, 1.29) |  | 103805 | 484753 | 7812 | 6004.8 | 1.30 (1.27, 1.33) |
| 2016 |  | 108178 | 418102 | 10018 | 7693.9 | 1.30 (1.28, 1.33) |  | 103741 | 418598 | 6847 | 5209.4 | 1.31 (1.28, 1.35) |
| 2017 |  | 110024 | 358646 | 8738 | 6583.4 | 1.33 (1.30, 1.36) |  | 103275 | 348770 | 5834 | 4310.3 | 1.35 (1.32, 1.39) |
| 2018 |  | 109692 | 285964 | 7012 | 5187.6 | 1.35 (1.32, 1.38) |  | 102407 | 275269 | 4693 | 3388.1 | 1.39 (1.35, 1.43) |
| 2019 |  | 112736 | 215082 | 5430 | 3847.4 | 1.41 (1.37, 1.45) |  | 103513 | 201667 | 3613 | 2440.5 | 1.48 (1.43, 1.53) |
| 2020 |  | 100683 | 113769 | 3505 | 2007.8 | 1.75 (1.69, 1.80) |  | 92796 | 106202 | 2123 | 1264.2 | 1.68 (1.61, 1.75) |
| 2000 | Non-Hispanic Black | 12981 | 113164 | 2108 | 2653.3 | 0.79 (0.76, 0.83) |  | 10765 | 96718 | 1523 | 1057.6 | 1.44 (1.37, 1.51) |
| 2001 |  | 13732 | 118924 | 2303 | 2742.8 | 0.84 (0.81, 0.87) |  | 11129 | 98717 | 1560 | 1081.0 | 1.44 (1.37, 1.52) |
| 2002 |  | 13774 | 121519 | 2329 | 2747.8 | 0.85 (0.81, 0.88) |  | 11596 | 102048 | 1668 | 1123.8 | 1.48 (1.41, 1.56) |
| 2003 |  | 13716 | 115715 | 2238 | 2563.1 | 0.87 (0.84, 0.91) |  | 11740 | 99687 | 1582 | 1083.9 | 1.46 (1.39, 1.53) |
| 2004 |  | 14023 | 117368 | 2159 | 2555.8 | 0.84 (0.81, 0.88) |  | 12093 | 101461 | 1702 | 1095.2 | 1.55 (1.48, 1.63) |
| 2005 |  | 13979 | 113830 | 2103 | 2419.5 | 0.87 (0.83, 0.91) |  | 12125 | 99073 | 1556 | 1070.8 | 1.45 (1.38, 1.53) |
| 2006 |  | 14586 | 118904 | 2242 | 2541.5 | 0.88 (0.85, 0.92) |  | 12364 | 98452 | 1638 | 1070.3 | 1.53 (1.46, 1.61) |
| 2007 |  | 15334 | 121056 | 2230 | 2524.5 | 0.88 (0.85, 0.92) |  | 13005 | 99607 | 1595 | 1080.2 | 1.48 (1.41, 1.55) |
| 2008 |  | 15624 | 118077 | 2190 | 2378.7 | 0.92 (0.88, 0.96) |  | 13013 | 94165 | 1507 | 1020.3 | 1.48 (1.40, 1.55) |
| 2009 |  | 16175 | 117263 | 2111 | 2316.7 | 0.91 (0.87, 0.95) |  | 14025 | 99417 | 1573 | 1075.7 | 1.46 (1.39, 1.54) |
| 2010 |  | 16063 | 109527 | 1906 | 2125.8 | 0.90 (0.86, 0.94) |  | 13965 | 92524 | 1440 | 992.7 | 1.45 (1.38, 1.53) |
| 2011 |  | 16093 | 103797 | 1878 | 1989.7 | 0.94 (0.90, 0.99) |  | 14513 | 90410 | 1460 | 967.4 | 1.51 (1.43, 1.59) |
| 2012 |  | 16087 | 92377 | 1660 | 1735.7 | 0.96 (0.91, 1.00) |  | 15027 | 87096 | 1328 | 928.8 | 1.43 (1.35, 1.51) |
| 2013 |  | 15850 | 84451 | 1657 | 1565.5 | 1.06 (1.01, 1.11) |  | 14968 | 79475 | 1264 | 847.6 | 1.49 (1.41, 1.58) |
| 2014 |  | 15805 | 75461 | 1441 | 1368.4 | 1.05 (1.00, 1.11) |  | 15511 | 75720 | 1147 | 793.3 | 1.45 (1.36, 1.53) |
| 2015 |  | 16422 | 70993 | 1316 | 1286.3 | 1.02 (0.97, 1.08) |  | 15829 | 68734 | 1035 | 725.0 | 1.43 (1.34, 1.52) |
| 2016 |  | 17041 | 64506 | 1274 | 1161.2 | 1.10 (1.04, 1.16) |  | 16250 | 61395 | 896 | 642.4 | 1.39 (1.30, 1.49) |
| 2017 |  | 17703 | 56306 | 1079 | 1007.5 | 1.07 (1.01, 1.14) |  | 16476 | 52430 | 788 | 546.2 | 1.44 (1.34, 1.55) |
| 2018 |  | 17726 | 45220 | 911 | 800.7 | 1.14 (1.07, 1.21) |  | 16661 | 42575 | 667 | 441.0 | 1.51 (1.40, 1.63) |
| 2019 |  | 18510 | 34494 | 756 | 598.1 | 1.26 (1.18, 1.36) |  | 17189 | 32228 | 542 | 328.3 | 1.65 (1.52, 1.80) |
| 2020 |  | 16510 | 18290 | 458 | 312.2 | 1.47 (1.34, 1.61) |  | 15686 | 17332 | 294 | 170.3 | 1.73 (1.53, 1.94) |
| 2000 | Non-Hispanic American Indian/Alaskan Native | 286 | 2575 | 57 | 30.1 | 1.89 (1.43, 2.45) |  | 351 | 3777 | 43 | 29.6 | 1.45 (1.05, 1.96) |
| 2001 |  | 377 | 3544 | 56 | 40.7 | 1.38 (1.04, 1.79) |  | 409 | 4140 | 55 | 31.1 | 1.77 (1.33, 2.31) |
| 2002 |  | 374 | 3546 | 57 | 39.7 | 1.44 (1.09, 1.86) |  | 354 | 3844 | 42 | 30.1 | 1.40 (1.01, 1.89) |
| 2003 |  | 387 | 3285 | 61 | 39.8 | 1.53 (1.17, 1.97) |  | 410 | 3923 | 65 | 31.3 | 2.08 (1.60, 2.65) |
| 2004 |  | 381 | 3019 | 54 | 34.8 | 1.55 (1.17, 2.03) |  | 435 | 3930 | 47 | 31.4 | 1.50 (1.10, 1.99) |
| 2005 |  | 410 | 3489 | 61 | 45.2 | 1.35 (1.03, 1.73) |  | 420 | 3856 | 58 | 30.1 | 1.92 (1.46, 2.49) |
| 2006 |  | 429 | 3491 | 72 | 44.1 | 1.63 (1.28, 2.06) |  | 466 | 3972 | 60 | 31.6 | 1.90 (1.45, 2.44) |
| 2007 |  | 465 | 3479 | 63 | 41.3 | 1.52 (1.17, 1.95) |  | 452 | 3728 | 49 | 30.1 | 1.63 (1.20, 2.15) |
| 2008 |  | 457 | 3305 | 57 | 40.2 | 1.42 (1.07, 1.84) |  | 540 | 4216 | 60 | 35.5 | 1.69 (1.29, 2.18) |
| 2009 |  | 505 | 3491 | 57 | 42.8 | 1.33 (1.01, 1.73) |  | 524 | 4136 | 70 | 35.0 | 2.00 (1.56, 2.52) |
| 2010 |  | 496 | 2973 | 69 | 35.8 | 1.93 (1.50, 2.44) |  | 568 | 4010 | 59 | 33.7 | 1.75 (1.33, 2.26) |
| 2011 |  | 524 | 2903 | 55 | 35.1 | 1.57 (1.18, 2.04) |  | 571 | 3994 | 63 | 34.4 | 1.83 (1.41, 2.34) |
| 2012 |  | 481 | 2828 | 58 | 33.5 | 1.73 (1.32, 2.24) |  | 594 | 3703 | 40 | 31.6 | 1.26 (0.90, 1.72) |
| 2013 |  | 532 | 2699 | 55 | 31.7 | 1.74 (1.31, 2.26) |  | 615 | 3368 | 54 | 29.4 | 1.84 (1.38, 2.40) |
| 2014 |  | 565 | 2409 | 42 | 26.1 | 1.61 (1.16, 2.17) |  | 623 | 3205 | 37 | 27.0 | 1.37 (0.97, 1.89) |
| 2015 |  | 556 | 2092 | 52 | 22.7 | 2.29 (1.71, 3.01) |  | 674 | 2963 | 49 | 25.7 | 1.90 (1.41, 2.52) |
| 2016 |  | 604 | 2177 | 40 | 25.0 | 1.60 (1.15, 2.18) |  | 693 | 2598 | 36 | 20.9 | 1.72 (1.21, 2.39) |
| 2017 |  | 572 | 1643 | 34 | 19.3 | 1.76 (1.22, 2.46) |  | 698 | 2284 | 33 | 19.1 | 1.73 (1.19, 2.42) |
| 2018 |  | 582 | 1404 | 36 | 14.5 | 2.48 (1.74, 3.43) |  | 731 | 1854 | 26 | 15.2 | 1.71 (1.12, 2.51) |
| 2019 |  | 630 | 1114 | 29 | 12.6 | 2.29 (1.54, 3.30) |  | 714 | 1342 | 25 | 11.0 | 2.27 (1.47, 3.35) |
| 2020 |  | 604 | 645 | 25 | 7.3 | 3.42 (2.22, 5.05) |  | 692 | 761 | 8 | 6.2 | 1.29 (0.56, 2.54) |
| 2000 | Non-Hispanic Asian and Pacific Islanders | 6776 | 60593 | 931 | 898.8 | 1.04 (0.97, 1.10) |  | 7143 | 82848 | 1022 | 675.7 | 1.51 (1.42, 1.61) |
| 2001 |  | 7286 | 66303 | 1008 | 952.7 | 1.06 (0.99, 1.13) |  | 7897 | 89756 | 1016 | 731.2 | 1.39 (1.31, 1.48) |
| 2002 |  | 7590 | 67221 | 1103 | 969.2 | 1.14 (1.07, 1.21) |  | 8272 | 92261 | 1105 | 738.8 | 1.50 (1.41, 1.59) |
| 2003 |  | 7784 | 66197 | 1052 | 936.7 | 1.12 (1.06, 1.19) |  | 8252 | 88725 | 1034 | 707.1 | 1.46 (1.37, 1.55) |
| 2004 |  | 8025 | 68052 | 1082 | 925.4 | 1.17 (1.10, 1.24) |  | 8749 | 90541 | 1114 | 715.4 | 1.56 (1.47, 1.65) |
| 2005 |  | 8019 | 66041 | 1060 | 866.4 | 1.22 (1.15, 1.30) |  | 9183 | 92517 | 1160 | 723.2 | 1.60 (1.51, 1.70) |
| 2006 |  | 8475 | 66745 | 1095 | 871.7 | 1.26 (1.18, 1.33) |  | 9461 | 89576 | 1087 | 695.3 | 1.56 (1.47, 1.66) |
| 2007 |  | 9057 | 71240 | 1108 | 903.3 | 1.23 (1.16, 1.30) |  | 10040 | 91384 | 1073 | 717.1 | 1.50 (1.41, 1.59) |
| 2008 |  | 9163 | 67316 | 1104 | 814.9 | 1.35 (1.28, 1.44) |  | 10647 | 92298 | 1049 | 716.2 | 1.46 (1.38, 1.56) |
| 2009 |  | 9303 | 65286 | 983 | 780.4 | 1.26 (1.18, 1.34) |  | 10900 | 88311 | 1025 | 673.8 | 1.52 (1.43, 1.62) |
| 2010 |  | 9556 | 61968 | 995 | 731.0 | 1.36 (1.28, 1.45) |  | 11304 | 85978 | 983 | 657.0 | 1.50 (1.40, 1.59) |
| 2011 |  | 9911 | 60694 | 997 | 704.2 | 1.42 (1.33, 1.51) |  | 11687 | 83164 | 966 | 628.9 | 1.54 (1.44, 1.64) |
| 2012 |  | 9622 | 54366 | 854 | 605.5 | 1.41 (1.32, 1.51) |  | 12153 | 79490 | 963 | 602.1 | 1.60 (1.50, 1.70) |
| 2013 |  | 10102 | 51675 | 834 | 557.6 | 1.50 (1.40, 1.60) |  | 12704 | 75019 | 915 | 561.7 | 1.63 (1.53, 1.74) |
| 2014 |  | 10151 | 46919 | 731 | 487.8 | 1.50 (1.39, 1.61) |  | 12996 | 69155 | 842 | 513.5 | 1.64 (1.53, 1.75) |
| 2015 |  | 10652 | 43855 | 725 | 454.0 | 1.60 (1.48, 1.72) |  | 13775 | 64982 | 758 | 479.3 | 1.58 (1.47, 1.70) |
| 2016 |  | 11019 | 39298 | 725 | 403.9 | 1.80 (1.67, 1.93) |  | 14130 | 56963 | 666 | 420.1 | 1.59 (1.47, 1.71) |
| 2017 |  | 11587 | 35307 | 589 | 361.3 | 1.63 (1.50, 1.77) |  | 14844 | 50071 | 595 | 368.1 | 1.62 (1.49, 1.75) |
| 2018 |  | 11638 | 28684 | 558 | 291.7 | 1.91 (1.76, 2.08) |  | 15084 | 40385 | 509 | 292.1 | 1.74 (1.59, 1.90) |
| 2019 |  | 11942 | 21357 | 374 | 211.0 | 1.77 (1.60, 1.96) |  | 15647 | 30003 | 384 | 212.9 | 1.80 (1.63, 1.99) |
| 2020 |  | 10797 | 11541 | 236 | 110.9 | 2.13 (1.87, 2.42) |  | 14206 | 16086 | 250 | 113.3 | 2.21 (1.94, 2.50) |
| 2000 | Hispanics | 10310 | 102408 | 1451 | 1536.2 | 0.94 (0.90, 0.99) |  | 10790 | 123528 | 1443 | 976.3 | 1.48 (1.40, 1.56) |
| 2001 |  | 11139 | 109760 | 1572 | 1595.4 | 0.99 (0.94, 1.04) |  | 11197 | 122996 | 1493 | 984.4 | 1.52 (1.44, 1.60) |
| 2002 |  | 11731 | 113469 | 1610 | 1652.6 | 0.97 (0.93, 1.02) |  | 11778 | 124063 | 1527 | 993.3 | 1.54 (1.46, 1.62) |
| 2003 |  | 11936 | 110396 | 1570 | 1572.0 | 1.00 (0.95, 1.05) |  | 12339 | 125686 | 1519 | 996.4 | 1.52 (1.45, 1.60) |
| 2004 |  | 12947 | 118374 | 1727 | 1646.7 | 1.05 (1.00, 1.10) |  | 12928 | 128143 | 1503 | 1017.2 | 1.48 (1.40, 1.55) |
| 2005 |  | 13323 | 115935 | 1616 | 1582.7 | 1.02 (0.97, 1.07) |  | 13622 | 131686 | 1572 | 1042.1 | 1.51 (1.43, 1.58) |
| 2006 |  | 13719 | 114908 | 1674 | 1535.7 | 1.09 (1.04, 1.14) |  | 13871 | 126072 | 1556 | 989.4 | 1.57 (1.50, 1.65) |
| 2007 |  | 14485 | 118145 | 1670 | 1549.6 | 1.08 (1.03, 1.13) |  | 14804 | 129961 | 1582 | 1012.2 | 1.56 (1.49, 1.64) |
| 2008 |  | 15138 | 116948 | 1636 | 1486.4 | 1.10 (1.05, 1.16) |  | 15813 | 131359 | 1599 | 1017.8 | 1.57 (1.49, 1.65) |
| 2009 |  | 15779 | 116486 | 1742 | 1445.7 | 1.20 (1.15, 1.26) |  | 16361 | 128285 | 1618 | 990.3 | 1.63 (1.56, 1.72) |
| 2010 |  | 15950 | 109006 | 1564 | 1320.4 | 1.18 (1.13, 1.24) |  | 16711 | 122714 | 1435 | 953.1 | 1.51 (1.43, 1.59) |
| 2011 |  | 16643 | 104645 | 1448 | 1246.1 | 1.16 (1.10, 1.22) |  | 17682 | 120506 | 1501 | 933.2 | 1.61 (1.53, 1.69) |
| 2012 |  | 16599 | 95884 | 1351 | 1091.1 | 1.24 (1.17, 1.31) |  | 18645 | 116941 | 1400 | 891.8 | 1.57 (1.49, 1.65) |
| 2013 |  | 16775 | 87795 | 1335 | 984.3 | 1.36 (1.28, 1.43) |  | 19208 | 108783 | 1310 | 837.4 | 1.56 (1.48, 1.65) |
| 2014 |  | 17413 | 82069 | 1246 | 880.7 | 1.41 (1.34, 1.50) |  | 20348 | 103716 | 1275 | 786.9 | 1.62 (1.53, 1.71) |
| 2015 |  | 18221 | 76358 | 1187 | 803.4 | 1.48 (1.39, 1.56) |  | 21764 | 97707 | 1206 | 737.8 | 1.63 (1.54, 1.73) |
| 2016 |  | 19003 | 68307 | 1063 | 716.4 | 1.48 (1.40, 1.58) |  | 22538 | 86690 | 1076 | 656.6 | 1.64 (1.54, 1.74) |
| 2017 |  | 19621 | 59340 | 959 | 613.7 | 1.56 (1.47, 1.66) |  | 22874 | 72597 | 901 | 544.1 | 1.66 (1.55, 1.77) |
| 2018 |  | 19707 | 47370 | 770 | 478.6 | 1.61 (1.50, 1.73) |  | 23529 | 59453 | 789 | 439.8 | 1.79 (1.67, 1.92) |
| 2019 |  | 20966 | 36804 | 659 | 361.0 | 1.83 (1.69, 1.97) |  | 24730 | 45280 | 643 | 328.5 | 1.96 (1.81, 2.11) |
| 2020 |  | 19263 | 20433 | 381 | 191.9 | 1.99 (1.79, 2.20) |  | 22270 | 24368 | 367 | 170.6 | 2.15 (1.94, 2.38) |
