# Supplementary material for: Rising Risk of Subsequent Primary Cancers Among US Cancer Survivors, 2000–2021
Source: Cancer Med. 2026 Apr 6;15(4):e71778. doi: 10.1002/cam4.71778 (PMC13052203; doi:10.1002/cam4.71778)
Supplement: Supplementary file 3 — Data S1: Supporting Information. [file CAM4-15-e71778-s001.docx]

**Supplementary information**

1. **Detailed estimates from Joinpoint regression for Figure 2:**

**Panel A. OER by Latency.** For females, Joinpoint identified three segments for latency periods of 2-5 months: an increase from 2000 to 2007 (APC = 4.6%, 95% CI: 3.9%, 6.6%), an increase from 2007 to 2013 (APC = 2.6%, 95% CI: 1.6%, 3.7%), and a non-significant trend from 2013 to 2020 (APC = 0.9%, 95% CI: –0.4%, 1.4%); one segment for latencies of 6–11 month: an increase from 2000 to 2020 (APC = 1.1%, 95% CI: 0.9%, 1.4%); two segments for latencies of 12–59 months: an increase from 2000 to 2008 (APC = 1.0%, 95% CI: 0.6%, 1.6%) and a decline from 2008 to 2016 (APC = –0.5%, 95% CI: –1.1%, –0.2%); two segments for latencies of 60–119 months: an increase from 2000 to 2006 (APC = 0.6%, 95% CI: 0.2%, 2.5%) and a declined from 2006 to 2011 (APC = –0.7%, 95% CI: –2.9%, –0.2%); one segment for latencies of 120+ months: a non-significant change (APC = 0.3%, 95% CI: –0.2%, 0.7%). For males, Joinpoint regression identified: two segements for latencies of 2–5 months: an increase from 2000 to 2014 (APC = 3.0%, 95% CI: 2.6%, 4.0%) and a non-significant trend from 2014 to 2020 (APC = 0.4%, 95% CI: –3.5%, 1.8%); one segment for latencies of 6–11 months: an increase from 2000 to 2020 (APC = 2.5%, 95% CI: 2.1%, 2.9%); two segments for latencies of 12–59 months: an increase from 2000 to 2013 (APC = 1.8%, 95% CI: 1.6%, 4.6%) and a non-significant trend from 2014 to 2016 (APC = -0.2%, 95% CI: -3.4%, 1.5%); two segments for latencies of 60–119 months: an increase from 2000 to 2005 (APC = 1.4%, 95% CI: 0.8%, 3.2%) and a non-significant trend afterwards (APC = 0.1%, 95% CI:- 1.5%, 0.6%); two segments for latencies of 120+ months, non-significant trend from 2000 to 2009 (APC < 0.1%, 95% CI: –1.2%, 1.1%) and another non-significant trend from 2009 to 2011 (APC = 5.6%, 95% CI: –0.5%, 8.9%).

**Panel B. OER by Site of the Index Cancer.** For females, Joinpoint regression identified three segments for colorectal cancer: two non-significant trends from 2000–2002 (APC = –2.2%, 95% CI: –4.0%, 1.7%) and 2002–2018 (APC = 1.8%, 95% CI: –1.1%, 3.7%), and an increase from 2018–2020 (APC = 11.6%, 95% CI: 1.7%, 16.2%). Two segments for lung & bronchus cancer: an increase from 2000–2013 (APC = 1.5%, 95% CI: 0.9%, 3.9%) followed by a non-significant trend from 2013–2020 (APC = –1.2%, 95% CI: –8.4%, 0.5%). Two segments for melanoma: an increase from 2000–2017 (APC = 1.7%, 95% CI: 0.8%, 2.0%) followed by another increase from 2017–2020 (APC = 8.3%, 95% CI: 2.1%, 15.4%). Four segments for breast cancer: a non-significant trend from 2000–2005 (APC = –0.1%, 95% CI: –0.7%, 0.8%), a decline from 2005–2015 (APC = –1.7%, 95% CI: –3.1%, –0.7%), a non-significant trend from 2015–2018 (APC = 2.0%, 95% CI: –2.6%, 3.4%), and an increase from 2018–2020 (APC = 14.2%, 95% CI: 6.7%, 18.7%). Two segments for uterus cancer: an increase from 2000–2017 (APC = 1.3%, 95% CI: 0.9%, 1.6%) and a sharper increase from 2017–2020 (APC = 11.1%, 95% CI: 4.3%, 20.5%). For males, Joinpoint regression idenfied 3 segments for colorectal cancer: two non-signifcant trends from 2000–2002 (APC = –2.2%, 95% CI: –4.0%, 1.7%) and 2002–2018 (APC = 1.8%, 95% CI: –1.1%, 3.7%) and a significant increase from 2018–2020 (APC = 11.6%, 95% CI: 1.7%, 16.2%). Lung & bronchus cancers: two segments were identified: an increase from 2000–2013 (APC = 1.5%, 95% CI: 0.9%, 3.9%) and a non-significant trend from 2013–2020 (APC = –1.2%, 95% CI: –8.4%, 0.5%). Melanoma: two segments were identified: an increase from 2000–2017 (APC = 1.7%, 95% CI: 0.8%, 2.0%) and another increase from 2017–2020 (APC = 8.3%, 95% CI: 2.1%, 15.4%). Prostate cancer: three segments were identifed. Two non-significant trends from 2000–2012 (APC = –0.1%, 95% CI: –0.9%, 0.7%) and 2012–2018 (APC = 1.1%, 95% CI: –0.3%, 2.7%) and an increase from 2018–2020 (APC = 9.4%, 95% CI: 2.4%, 12.9%). Urinary Bladder cancer: three progressive increases were identified for 2000–2015 (APC = 2.1%, 95% CI: 1.2%, 2.8%), 2015–2018 (APC = 7.0%, 95% CI: 1.7%, 8.7%), and 2018–2020 (APC = 22.7%, 95% CI: 13.7%, 28.9%).

**Panel C. OER by Stage of the Index Cancer**. For females, Joinpoint regression identified two segments for localized cancers: a non-significant trend from 2004–2016 (APC = 0.2%, 95% CI: –0.3%, 0.5%) followed by an increase from 2016–2020 (APC = 5.2%, 95% CI: 2.3%, 11.3%); two segments for regional cancers: an increase from 2004–2017 (APC = 0.4%, 95% CI: 0.1%, 0.7%) and a sharper increase from 2017–2020 (APC = 9.6%, 95% CI: 5.3%, 16.2%); three segments for distant cancers: an increase from 2004–2012 (APC = 2.0%, 95% CI: 1.3%, 4.3%), a non-significant trend from 2012–2017 (APC = –0.7%, 95% CI: –4.1%, 0.7%), and an increase from 2017–2020 (APC = 7.8%, 95% CI: 2.9%, 14.5%). For males, Joinpoint regression identified: Two segments for localized cancers: a non-significant trend from 2004–2011 (APC = 1.0%, 95% CI: –0.5%, 1.6%) followed by an increase from 2011–2020 (APC = 3.6%, 95% CI: 2.8%, 5.0%); two segments for regional cancers: an increase from 2004–2018 (APC = 1.9%, 95% CI: 0.8%, 2.4%) followed by a sharper increase from 2018–2020 (APC = 14.5%, 95% CI: 2.8%, 19.3%); three segments for distant cancers: an increase from 2004–2011 (APC = 2.8%, 95% CI: 1.8%, 4.3%), a non-significant trend from 2011–2018 (APC = –0.1%, 95% CI: –3.2%, 1.2%), and an increase from 2018–2020 (APC = 8.2%, 95% CI: 0.7%, 12.2%).

1. **Detailed estimates from Joinpoint regression for Figure 3**

**Panel A. OER by Age at Diagnosis of the Index Cancer**. For females, Joinpoint regression identified one segment for ages 0–14: a significant increase from 2000–2020 (APC = 6.1%, 95% CI: 3.6%, 8.0%); two segments for ages 15–39: a non-significant trend from 2000–2013 (APC = 2.2%, 95% CI: –1.6%, 3.1%), and a significant increase from 2013–2020 (APC = 7.4%, 95% CI: 3.2%, 21.7%); two segments for ages 40–49: a significant increase from 2000–2017 (APC = 1.0%, 95% CI: 0.6%, 1.3%) and a sharp increase from 2017–2020 (APC = 15.8%, 95% CI: 6.8%, 28.9%); three segments for ages 50–64: a significant increase from 2000–2014 (APC = 0.6%, 95% CI: 0.3%, 0.7%), an increase from 2014–2018 (APC = 2.9%, 95% CI: 0.8%, 4.3%), and an increase from 2018–2020 (APC = 12.9%, 95% CI: 7.4%, 15.7%); four segments for ages 65–84: three non-significant trend from 2000–2002 (APC = –0.2%, 95% CI: –1.3%, 1.6%), 2002–2006 (APC = 2.1%, 95% CI: –0.5%, 3.2%) and 2006–2017 (APC = 0.3%, 95% CI: –1.1%, 0.9%), followed by and a significant increase from 2017–2020 (APC = 5.6%, 95% CI: 3.0%, 9.3%); two segments for ages 85+: a significant increase from 2000–2018 (APC = 1.8%, 95% CI: 1.0%, 2.2%) and a sharp increase from 2018–2020 (APC = 12.2%, 95% CI: 2.4%, 18.4%); three segments for ages 85+: a significant increase from 2000–2010 (APC = 2.7%, 95% CI: 2.1%, 5.6%), a non-significant trend from 2010–2017 (APC = 0.6%, 95% CI: –3.2%, 1.7%), and a significant increase from 2017–2020 (APC = 7.8%, 95% CI: 2.6%, 14.8%).

For males, Joinpoint regression identified one segment for ages 0–14: a significant increase from 2000–2020 (APC = 5.4%, 95% CI: 2.6%, 7.6%); one segment for ages 15–39: a significant increase from 2000–2020 (APC = 6.1%, 95% CI: 5.0%, 7.0%); three segments for ages 40–49: a non-significant trend from 2000–2004 (APC = 0.8%, 95% CI: –3.9%, 4.2%), a significant increase from 2004–2012 (APC = 4.6%, 95% CI: 0.8%, 13.2%), and a non-significant trend from 2012–2020 (APC = 9.7%, 95% CI: –7.8%, 20.7%); three segments for ages 50–64: a significant increase from 2000–2009 (APC = 1.6%, 95% CI: 1.0%, 1.9%), another significant increase from 2009–2018 (APC = 3.7%, 95% CI: 3.1%, 4.5%), and a sharp rise from 2018–2020 (APC = 18.7%, 95% CI: 11.3%, 23.6%); three segments for ages 65–84: a non-significant trend from 2000–2007 (APC = 0.9%, 95% CI: –0.3%, 1.5%), a significant increase from 2007–2018 (APC = 2.2%, 95% CI: 1.5%, 2.7%), and a sharper increase from 2018–2020 (APC = 8.0%, 95% CI: 2.6%, 10.6%).

**Panel B. OER by Race/Ethnicity**. NH: Non-Hispanic. AI/AN: American Indian or Alaskan Native. API: Asian and Pacific Islanders. For females, Joinpoint regression identified two segments for NH White individuals: a significant increase from 2000–2017 (APC = 0.7%, 95% CI: 0.5%, 0.8%) and an increase from 2017–2020 (APC = 7.3%, 95% CI: 4.4%, 11.9%); three segments for NH Black individuals: a significant increase from 2000–2004 (APC = 1.3%, 95% CI: 0.0%, 4.6%), a significant decline from 2004–2017 (APC = –0.4%, 95% CI: –3.0%, –0.2%), and a sharp increase from 2017–2020 (APC = 7.0%, 95% CI: 2.0%, 13.8%); one segment for NH AI/AN individuals: a non-significant trend from 2000–2020 (APC = 0.2%, 95% CI: –1.6%, 1.7%); two segments for NH API individuals: a non-significant trend from 2000–2017 (APC = 0.6%, 95% CI: –1.2%, 1.5%) followed by a significant increase from 2017–2020 (APC = 8.6%, 95% CI: 0.7%, 17.9%); two segments for Hispanic individuals: a significant increase from 2000–2017 (APC = 0.5%, 95% CI: 0.3%, 0.7%) followed by a sharper increase from 2017–2020 (APC = 9.4%, 95% CI: 5.5%, 16.0%).

For males, Joinpoint regression identified three segments for NH White individuals: a significant increase from 2000–2007 (APC = 1.1%, 95% CI: 0.2%, 1.5%), an increase from 2007–2018 (APC = 2.6%, 95% CI: 2.1%, 3.0%), and a sharper rise from 2018–2020 (APC = 10.4%, 95% CI: 3.9%, 13.8%); three segments for NH Black individuals: two non-significant trends from 2000–2010 (APC = 1.3%, 95% CI: –0.9%, 4.1%) and 2010–2018 (APC = 2.6%, 95% CI: –0.7%, 4.4%), followed by a significant increase from 2018–2020 (APC = 12.8%, 95% CI: 3.2%, 18.3%); two segments for NH AI/AN: a non-significant trend from 2000–2017 (APC = 1.2%, 95% CI: –3.1%, 2.5%) and a significant increase from 2017–2020 (APC = 21.7%, 95% CI: 3.0%, 55.3%); one segment for NH API individuals: a significant increase from 2000–2020 (APC = 3.0%, 95% CI: 2.5%, 3.5%); two segments for Hispanic individuals: a non-significant trend from 2000–2011 (APC = 2.2%, 95% CI: –0.2%, 2.9%) and a significant increase from 2011–2020 (APC = 4.9%, 95% CI: 3.6%, 9.2%).
